# Natural Antimicrobial Activity of Nettle ( *Urtica dioica* L.) Leaf Extract for Shelf‐Life Extension of Mashed Potatoes

**DOI:** 10.1002/fsn3.71551

**Published:** 2026-02-24

**Authors:** Saritha Kagula, Steven Harte, Srivathsa Kumbaji, Rania Harastani, Mary Nkongho Tanyitiku

**Affiliations:** ^1^ Food and Markets Department, Natural Resources Institute University of Greenwich Kent UK; ^2^ Agriculture, Health and Environment Department, Natural Resources Institute University of Greenwich Kent UK; ^3^ Medway Food Innovation Centre, Natural Resources Institute University of Greenwich Kent UK

**Keywords:** antimicrobial activity, mashed potatoes, natural antimicrobials, nettle extract, nisin, shelf‐life extension

## Abstract

The growing demand for minimally processed clean‐label foods has intensified interest in natural antimicrobials as alternatives to synthetic preservatives. However, very little is known about the antimicrobial potential of several wild edible plants when incorporated into food matrices. This study evaluated the antimicrobial activity of nettle ( *Urtica dioica* L.) leaf extract and as a clean‐label preservative for extending the shelf life of fresh mashed potatoes. The extract exhibited strong antioxidant activity, with DPPH, ABTS, and FRAP values of 21.96 ± 0.76 μmol Trolox/mL, 17.51 ± 0.90 μmol Trolox/mL, and 5.93 ± 0.65 Fe(II)/g, respectively. In vitro antimicrobial testing confirmed broad‐spectrum activity, with minimum inhibitory and bactericidal concentrations indicating pronounced susceptibility of Gram‐positive bacteria (*Staphylococcus aureus, Bacillus cereus*, *Listeria monocytogenes* ) and notable effects on Gram‐negative pathogens (*E. coli, Salmonella enterica* serovar *Typhimurium*). Cytotoxicity assessment using L929 fibroblast cells showed the extract was non‐toxic at concentrations effective for antimicrobial application. When incorporated into mashed potatoes at 0.5%–2.0% (w/v), nettle extract achieved preservative effects comparable to 0.025% commercial nisin. Treated samples exhibited significantly delayed increases in total viable counts, psychrotrophs, Enterobacteriaceae, *B. cereus, S* *. aureus* *, P. aeruginosa*, and spoilage fungi during storage at 4°C and 25°C. Electronic tongue analysis differentiated treatment groups, revealing mild bitterness and astringency at increasing nettle leaf extract incorporation, but these effects were less detrimental than spoilage‐related off‐flavors in untreated controls. Overall, nettle leaf extract provides combined antimicrobial and antioxidant functionality, enhances microbial stability, and maintains acceptable sensory quality, supporting its potential as a natural alternative to synthetic preservatives in ready‐to‐eat mashed potato products.

## Graphical abstract

NLE exhibited strong antioxidant activity, with DPPH, ABTS, and FRAP values of 21.96 ± 0.76 and 17.51 ± 0.90 μmol Trolox/mL, and 5.93 ± 0.65 μmol Fe (II)/g, respectively. Cytotoxicity assessment using L929 fibroblast cells indicated that the extract was non‐toxic at concentrations relevant for antimicrobial application. In vitro antimicrobial assays demonstrated broad‐spectrum activity, with the highest selectivity observed against *L. monocytogenes* (SI = 53.36) and *P. aeruginosa* (SI = 63.54), two organisms of major importance in the spoilage and safety of refrigerated mashed potatoes.

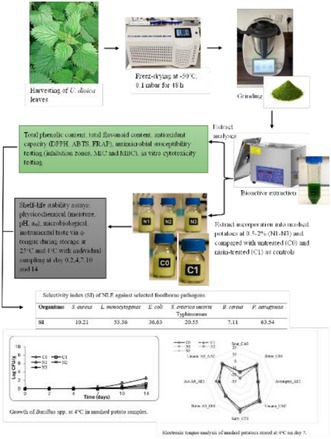

## 1 Introduction

The growing demand for clean‐label and sustainable food products has driven the food industry to seek alternatives to synthetic preservatives. Natural plant extracts, rich in bioactive compounds with antimicrobial and antioxidant properties, are increasingly regarded as viable solutions for extending shelf life while meeting consumer expectations for food safety (Karnwal and Malik 2024; Mei et al. 2019; Zhao et al. 2022). These extracts not only inhibit microbial growth and oxidative deterioration but also align with global trends toward environmentally responsible and health‐conscious food systems (Karnwal and Malik 2024; Petcu et al. 2023). Among the diverse sources of plant‐based bioactives, wild edible plants offer unique opportunities as natural antimicrobials (Alirezalu et al. 2021; Arfa et al. 2023; Gülhan and Yangilar 2024; Harrison et al. 2022).

Stinging nettle ( *Urtica dioica* L.) has gained attention due to its long history of culinary and medicinal use and its rich composition of phenolic acids (e.g., caffeic, chlorogenic), flavonoids (e.g., kaempferol, quercetin), organic acids, and other secondary metabolites (Kregiel et al. 2018; Mahmoudi et al. 2014; Sahal et al. 2025; Zhang et al. 2023). For instance, tincture, a nettle alcoholic extract, is becoming increasingly popular mainly because they allow better availability of phytochemicals and are stable over a longer period (Šic Žlabur et al. 2022). In addition, previous studies have demonstrated that these nettle extracts, prepared using solvents such as hexane, ethyl acetate, ethanol, and water, exhibit inhibitory effects against a wide range of microorganisms, including Gram‐positive and Gram‐negative bacteria, yeasts, and filamentous fungi (Ahmadi et al. 2014; Elez Garofulić et al. 2021; Mahmoudi et al. 2014; Modarresi‐Chahardehi et al. 2012). While the in vitro antimicrobial potential of nettle extract is well‐studied, its practical efficacy and sensory impact in real food systems remain underexplored. These gaps are particularly critical for highly perishable foods such as mashed potatoes, which are prone to rapid microbial spoilage, enzymatic degradation, and quality deterioration during storage (CIP 2024; Doan and Davidson 2000; Yu et al. 2024).

Mashed potatoes are a culinary preparation made from cooked potatoes that are crushed into a smooth or semi‐smooth consistency, usually with added ingredients such as milk and herbs for flavor and texture. Their nutritional composition includes approximately 79% water content, 2.2 g/100 g protein, 1.5–2.2 g/100 g fat, and 13.9–14.6 g/100 g carbohydrate (Cao et al. 2025). Just like other potato products, mashed potatoes, whether freshly prepared or reconstituted from dehydrated flakes, are highly perishable (shelf‐life ca. 7 days), owing to this high moisture content, rich nutrient profile, neutral to slightly alkaline pH, that favor the growth of spoilage organisms and opportunistic pathogens including *Bacillus cereus* , *Pseudomonas* spp., *Listeria monocytogenes* , and Enterobacteriaceae (do Nascimento et al. 2020; Thomas et al. 2002; Xu et al. 2023). As a result, refrigerated potato and mashed potato products often exhibit limited shelf life unless preservatives or antimicrobial processing hurdles are applied (Thomas et al. 2002; Zhao et al. 2022). Given rising consumer concerns about synthetic additives, natural plant‐based antimicrobials such as nettle extract offer a promising alternative for extending shelf life while maintaining clean‐label status.

Therefore, this study aims to address this gap by evaluating the antimicrobial activity of nettle leaf extract and assessing its potential in enhancing the microbial stability and quality of mashed potatoes under realistic storage conditions. In light of the strong bioactive profile of *U. dioica* and the clear need for natural preservation strategies in ready‐to‐eat starchy foods, there is scientific and industrial value in systematically evaluating the antimicrobial, antioxidant, and sensory effects of nettle leaf extracts in mashed potato products.

## 2 Materials and Methods

### 2.1 Materials

Fresh leaves of *Urtica dioica* L. were obtained from a vegetable farm in Dockside, Chatham, United Kingdom, identified by their morphological characteristics (Tanyitiku et al. 2024). The leaves were washed thoroughly under running deionized water, drained and freeze‐dried at −50°C, 0.1 mbar, 48 h using a FT33 MK11 vacuum freeze dryer (Armfield, United Kingdom) for 48 h. It was ground to a fine powder using a Thermomix TM6 (Vorwerk, Wuppertal, Germany), passed through a < 250 μm UWL 400 T sieve shaker (Haver & Boecker, VWR International, United Kingdom) and then stored in airtight polyethylene bags during analysis. Maris Piper fresh potatoes were purchased from a local supermarket (Sainsbury's, Chatham, United Kingdom). Unless stated, chemicals and reagents were obtained from Merck Life Science (Sigma‐Aldrich brand, Gillingham, United Kingdom) and were of analytical grade. All culture media were obtained from Oxoid Ltd., Basingstoke, Hampshire, United Kingdom.

### 2.2 Nettle Leaf Extraction

Nettle leaf extract (NLE) was prepared following a combined ultrasonication‐maceration procedure (Šic Žlabur et al. 2022). Here, 1 g of dried nettle leaf powder was mixed with 10 mL of deionized water in a sterile glass tube. The mixture was first subjected to ultrasonic treatment in a digital ultrasonic bath (Shenzhen Co. Ltd., Hong Kong, China) at 65°C for 30 min to enhance cell wall disruption and promote compound release. After sonication, the suspension was transferred to an Eppendorf Thermomixer Comfort (Hamburg, Germany) and macerated at 350 rpm and 25°C for 4 h. The extract was then centrifuged (Heraeus Megafuge 8 centrifuge, Thermo Scientific, United Kingdom) at 4000 × *g* for 10 min, and the supernatant was collected, filtered (0.45 μm), and stored at 4°C until further use. Notably, water was selected as the extraction solvent because aqueous extracts of fresh nettle leaves and roots were found to contain significantly higher total polyphenol contents with stronger antimicrobial activity than ethanolic extracts across multiple leaf harvest periods (Flórez et al. 2022; Kőszegi et al. 2019, 2023). Extracts were stored at −20°C until use.

The extraction yield of NLE was estimated according to Lohvina et al. (2022). In brief, a prepared filtrate was evaporated in a rotary evaporator at 50°C until a thick extract was obtained. The thick extract was then dried at 50°C for 24 h in an incubator (Gallenkamp, United Kingdom) and was then weighed. The extract yield was calculated as percent of initial weight to the dry weight and expressed as a percentage.

### 2.3 Preparation of Mashed Potato Samples

Freshly purchased potatoes were peeled, cut into 2–3 cm cubes, and boiled in deionized water for 10 min until fully cooked (fork penetrated without resistance). The cooked potatoes were then drained thoroughly, mashed manually using a sterile potato masher, and cooled to 25°C. Five treatments were then prepared: C0 (negative control): no preservative, C1 (positive control): 0.025% w/v (250 ppm) nisin from *Lactococcus lactis* (potency ≥ 900 IU/mg), a bacteriocin widely used in the food industry, N1 to N3 (nettle leaf extract incorporated mashed potatoes) where N1: 0.5% (w/v) nettle leaf extract, N2: 1.0% (w/v) nettle leaf extract, and N3: 2.0% (w/v) nettle leaf extract. Nisin was selected as a control preservative as it was affirmed as Generally Recognized as Safe (GRAS) for use as an antimicrobial agent, for instance at maximum level of 12 mg/kg in unripen cheese and 25 mg/kg in heat‐treated meat products (FDA 1988; Younes et al. 2017). In addition, nisin‐incorporated mashed potatoes at a concentration of 6.25 mg/g were reported to extend the product shelf life by at least 58 days compared to untreated controls (Thomas et al. 2002).

To understand the effects of NLE‐incorporated mashed potatoes during storage, portions (50 g) of freshly prepared mashed potatoes were aseptically transferred into sterile Simax glass bottles (100 mL capacity) and thoroughly mixed with each prepared treatment. Individual bottles were prepared for each sampling time point (0, 2, 4, 7, 10, and 14 days) to prevent cross‐contamination. Samples were stored under two conditions: refrigerated (4°C ± 1°C) and ambient (25°C ± 1°C). At each interval, one complete bottle per treatment was withdrawn for analyses.

### 2.4 Phytochemical Analysis of NLE

#### 2.4.1 Total Phenolic Content

The total phenolic content of nettle extracts was quantified using the Folin–Ciocalteu colorimetric method according to Lohvina et al. (2022). Briefly, 0.2 mL of the extract was mixed with 1.6 mL of deionized water, 0.3 mL of 20% (w/w) Na_2_CO_3_ and 0.1 mL of Folin–Ciocalteu reagent. It was kept in the dark under ambient conditions for 1 h to complete the reaction and was then diluted to 4 mL using distilled water. The absorbance was measured at 750 nm using a UV–VIS spectrophotometer (Jenway, Cole Palmer, United Kingdom). The calibration curve was established using gallic acid at concentrations of 0.01–0.1 mg/mL and the results of total phenolic content were expressed as mg of gallic acid equivalents per gram of extract (mg GAE/g extract).

#### 2.4.2 Total Flavonoid Content

Total flavonoid content was determined using the aluminium chloride colorimetric assay (Fattahi et al. 2014). In this procedure, aliquots of NLE were mixed with aluminium chloride solution, potassium acetate, and distilled water and incubated for 30 min at room temperature. The formation of a yellow‐colored complex was quantified spectrophotometrically at 415 nm. Quercetin was used to generate a standard calibration curve, and results were expressed as milligrams of quercetin equivalents (mg QE) per gram of extract.

#### 2.4.3 DPPH Radical Scavenging Activity

The antioxidant capacity was assessed using the DPPH (2,2‐diphenyl‐1‐picrylhydrazyl) radical scavenging assay (Gaber et al. 2023). Briefly, 0.2 mL of NLE was added to 2.8 mL of 0.1 mM DPPH solution in methanol solution, vortexed, and incubated in the dark at room temperature for 30 min. Absorbance was recorded at 517 nm, and the results were expressed as μmol Trolox Equivalent Antioxidant Capacity (TEAC) per mL.

#### 2.4.4 ABTS Radical Cation Scavenging Activity

The ABTS assay was carried out as described by Gaber et al. (2023). ABTS radical cation was dissolved at 7 mM in 2.45 mM potassium persulfate and allowed to stand in the dark for 16 h at 4°C to prepare the ABTS radical reagent solution. The resulting solution was diluted with 50 mM PBS (pH 7.4) to ensure an absorbance of 0.70 (±0.02) at 734 nm detected in a UV–vis spectrophotometer (Cary 60, Agilent Technologies, Santa Clara, CA, USA). Then 10 μL of each sample (at a final concentration of 1.0 mg/mL) was mixed with 990 μL of ABTS radical reagent solution; after 6 min incubation at room temperature and in the dark, the absorbance at 734 nm was read. Ascorbic acid (4 mM) was used as a positive control and PBS as a negative control. ABTS radical scavenging activity was expressed as TEAC (μmol Trolox/mL) of NLE against the standard curve (0.05–2 mM).

#### 2.4.5 Ferric Reducing Antioxidant Power (FRAP)

The ferric‐reducing activity of NLE was carried out as described by Thaipong et al. (2006). Here, 140 μL of each sample (at a final concentration of 2.27 mg/mL) was mixed with 140 μL of 200 mM PBS (pH 6.6) and 140 μL of potassium hexacyanoferrate (III) (10 mg/mL). The mixture was incubated at 50°C for 20 min, then the reaction was stopped by adding 200 μL of trichloroacetic acid (100 mg/mL) and then centrifuged at 2000 g for 10 min. 400 μL of supernatant was mixed with 400 μL of double distilled water and 80 μL of ferric chloride (1 mg/mL). This mixture was incubated in the dark for 10 min, and the absorbance was measured at 700 nm. BHT (20 mg/mL) was used as a positive control. Ferric reducing antioxidant power was expressed as μmol TEAC per mL of NLE.

### 2.5 In Vitro Cytotoxicity of NLE

Cell viability and proliferation were evaluated using the CyQUANT MTT Cell Proliferation Assay Kit (Thermo Fisher Scientific, Catalog No. V13154) following the manufacturer's protocol. Briefly, L929 mouse fibroblasts (ATCC CCL‐1) were maintained in Dulbecco's Modified Eagle Medium (DMEM, high glucose) supplemented with 10% fetal bovine serum and 1% penicillin‐streptomycin at 37°C in a humidified 5% CO_2_ atmosphere. Cells were seeded at 8 × 10^3^ cells/well in 96‐well plates and allowed to attach for 48 h. Nettle extract stocks (10 mg/mL) were prepared in 70% ethanol, sterile‐filtered and diluted in culture medium to final test concentrations of 25, 50, 100, 200, 400 and 800 μg/mL, ensuring the final solvent concentration did not exceed 1% v/v. Vehicle controls received medium containing the same solvent concentration. After incubation, 10 μL of a 12 mM MTT stock solution was added into each well. The negative control consisted of 10 μL of the MTT stock solution in 100 μL of the medium alone. The plates were incubated at 37°C for 3 h. Subsequently, the content in the wells was reduced to 25 μL of medium and 50 μL of DMSO to each well, then pipetted up and down thoroughly to mix. It was then incubated at 37°C for 10 min, thoroughly mixed by pipetting and absorbance was measured at 570 nm (Multiskan microplate photometer, Thermo Scientific, United Kingdom). Percent viability was calculated relative to vehicle controls.

In addition, the concentration‐response data were fitted to a four‐parameter logistic nonlinear regression model according to Equation (1).

(1) Y=Bottom+Top−Bottom1−CIC50^HillSlope

where Y represents cell viability (%), C is the extract concentration (μg/mL), Top and Bottom are the asymptotic maximum and minimum responses, respectively, and HillSlope describes the steepness of the curve. The IC_50_ value, defined as the concentration required to reduce cell viability by 50%, was calculated from the fitted model.

### 2.6 Antimicrobial Activity of NLE

The antimicrobial activity of NLE was assessed using the agar well diffusion method, according to CLSI guidelines. Six selected foodborne pathogens and spoilage organisms were used including *Staphylococcus aureus* NCTC 8532, *Listeria monocytogenes* NCTC 10357, *Escherichia coli* NCTC 9001, *Salmonella enterica* serovar Typhimurium NCTC 74, *Bacillus cereus* NCTC 2599, and *Pseudomonas aeruginosa* ATCC 9027. Strains were maintained at −80°C in glycerol stocks and sub‐cultured twice on tryptic soy agar (TSA) prior to experiments. Bacterial strains were grown overnight in Mueller‐Hinton broth (MHB) at 37°C and adjusted to approximately 10^6^ CFU/mL.

Sterile Mueller‐Hinton agar (MHA) plates were inoculated by lawn plating: 100 μL of the standardized bacterial suspension was spread evenly across the agar surface using a sterile swab and allowed to dry for 5 min. Sterile wells (~6 mm) were aseptically formed using sterile blue 1 mL pipette tips. Two drops of molten MHA were added to the base of the wells and allowed to dry for another 5 min. Subsequently, 100 μL of NLE solution (50 and 100 mg/mL) were added into the wells and allowed for another 10 min. Ciprofloxacin disc (CIP 5 μg) was included on each plate as a positive control to verify assay performance, and 100 μL sterile deionized water served as a negative control. Plates were incubated at 37°C for 24 h. After incubation, inhibition zone diameters (including the well diameter) were measured in millimeters using a digital caliper. Mean zone diameters ± standard deviation (*n* = 3) were obtained and interpreted by comparison of extract‐induced zones with the Ciprofloxacin 5 μg as positive control and water (negative) control. CLSI interpretive breakpoints (CLSI 2020) were consulted for the positive control antibiotic.

The minimum inhibitory concentration (MIC) was determined using the Microbial Viability Assay Kit (Dojindo Molecular Technologies, Kumamoto, Japan), according to manufacturer instructions and as slightly modified by Kramer et al. (2015). Here, two‐fold serial dilutions of NLE were prepared in sterile deionized water to yield final concentrations ranging from 0.125 to 64 mg/mL. Bacterial concentrations of approx. 10^6^CFU/mL were adjusted in twofold concentrated Mueller‐Hinton broth, and 100 μL of the test media containing the bacteria was combined in 100 μL of the appropriate dilution of the nettle extract in Greiner Bio‐One F‐Bottom Clear 96‐well microplates (VWR International, United Kingdom). The negative control was sterile deionized water, and sterile MHB served as a blank. After incubation at 37°C for 18 h, 10 μL of the coloring reagent was added; 1:10 (Gram‐negative bacteria: *E. coli*, *S. typhimurium* , *P. aeruginosa* ) or 1:80 (Gram‐positive bacteria: *S. aureus* , *L. monocytogenes, B. cereus*), and then incubated at 37°C for another 2 h. The absorbance was read at 450 nm using a Multiskan FC Type 357 Microplate Photometer (Thermo Scientific, China). Notably, the coloring reagent is a mixture of 9 parts of WST solution and 1 part of the electron mediator reagent, prepared according to the manufacturer's instructions.

To confirm bactericidal activity, 10 μL aliquots were aseptically taken from wells showing no visible growth in the MIC assay and spread‐plated onto Mueller‐Hinton agar. The plates were incubated at 37°C for 24 h. The MBC was defined as the lowest concentration of nettle extract resulting in no microbial colony growth on agar, indicating ≥ 99.9% microbial killing.

### 2.7 Selectivity Index

The selectivity index (SI) was used to assess the safety margin of NLE by comparing its cytotoxicity toward mammalian cells with its antimicrobial activity. SI was estimated as described by Famuyide et al. (2019), as the ratio between the extract concentration that caused a 50% reduction in cell viability (IC_50_) and the minimum inhibitory concentration (MIC) against each target microorganism (Equation 2).

(2) $$\mathrm{SI}=\frac{\mathrm{IC}50\;\left(\;\text{mammalian cells}\right)}{\mathrm{MIC}\;\left(\text{Bacteria}\right)}$$

where IC_50_ is the concentration of nettle leaf extract causing 50% inhibition of L929 fibroblast cell viability, and MIC is the minimum inhibitory concentration against each bacterial strain determined in the antimicrobial assays.

### 2.8 Shelf‐Life Stability Assay of NLE‐Incorporated Mashed Potatoes

#### 2.8.1 Moisture, pH and Water Activity

Moisture was determined directly using a CEM Smart 6 moisture analyzer (CEM Corp., Matthews, NC, USA) according to the AOAC 2008.06 official method (Leffler et al. 2008). For pH and water activity, samples were prepared according to Lauridsen and Knøchel (2003). Here, 10 g of each mashed potato sample was mixed with 10 g of sterile distilled water in a stomacher for 60 s. The pH of mashed potato samples was measured using a calibrated pH meter (Model 3510, Jenway, Stone, United Kingdom), with the electrode directly inserted into the homogenized samples at room temperature (25°C). Water activity (a_w_) was determined using the AQUALAB 4TE water activity meter (Pullman, USA).

#### 2.8.2 Microbiological Analysis

Mashed potato samples were analyzed for total aerobic bacteria, psychotropic (cold‐tolerant) bacteria, Enterobacteriaceae, *Staphylococcus aureus* , *Pseudomonas aeruginosa* , *Bacillus* spp., yeast and molds. Here, 10 g of each sample was aseptically homogenized with 90 mL of sterile Buffered Peptone Water using a Stomacher (Seward, Worthing, United Kingdom). Serial ten‐fold dilutions were prepared to 10^−6^ and plated on various agar media as follows. Total viable and psychotropic bacteria were enumerated on Plate Count Agar after incubation at 30°C for 48 h and 7°C ± 1°C for 10 days respectively. Enterobacteriaceae were enumerated on Violet Red Bile Glucose agar, incubated at 37°C for 24 h. Randomly selected colonies were then biochemically identified using API 20 strips. *Staphylococcus aureus* and *Pseudomonas aeruginosa* were enumerated on Baird‐Parker agar (including egg yolk tellurite) and cetrimide agar, respectively, after incubation at 37°C for 24 h. For heat‐tolerant *Bacillus* spores, the serial diluents were initially subjected to heat treatment at 80°C for 10 min in a water bath, pour‐plated onto PCA and incubated at 30°C for 48 h, allowing surviving spores to germinate and form colonies. Colonies displaying typical *Bacillus* morphology—large, dry, irregular, and opaque—were enumerated. Finally, yeasts and molds were enumerated using Dichloran Rose Bengal Chloramphenicol agar after incubation at 25°C for 5 days. Colony morphology was used to differentiate yeasts (typically smooth, moist colonies) from filamentous molds (fuzzy colonies).

#### 2.8.3 Instrumental Taste Evaluation

The evolution of taste attributes in treated mashed potato samples was assessed using an electronic tongue (TS‐5000Z, Intelligent Sensor Technology Inc., Kanagawa, Japan) at Day 0, 4, and 7 of the shelf‐life model experiments. Our choice of these sampling points was based on established spoilage kinetics of mashed potato products that reported these shelf‐life ranges (Doan and Davidson 2000; Thomas et al. 2002). Here, approximately 20 g of each sample was homogenized with 40 mL of deionized water using a Thermomix TM6 (Vorwerk, Wuppertal, Germany) for 2 min at 25°C to obtain a uniform slurry. The homogenate was centrifuged at 4000 × *g* for 15 min, and the clear supernatant was collected for analysis. The supernatants were placed in sample trays and loaded into the e‐tongue equipped with cross‐selective lipid membrane sensors designed to mimic human taste perception. We quantified taste attributes including sweetness, saltiness, sourness, bitterness, umami, and overall taste intensity. Prior to analysis, the e‐tongue system was calibrated with standard taste solutions to ensure accurate discrimination of each taste modality. Between measurements, the sensors were rinsed with especial washing solution and standard solutions and reconditioned in reference solution to prevent carryover effects.

### 2.9 Statistical Analysis

All analyses were carried out in triplicate for each treatment group and sampling interval. Data were analyzed using IBM SPSS Statistics using IBM SPSS v.31.0.0.0 (117) (IBM Corp., Armonk, NY, USA). The results are reported as mean ± standard deviation. One‐way analysis of variance (ANOVA) was performed to evaluate the effect of flour substitution levels on all measured parameters. When ANOVA indicated significant differences (*p* < 0.05), Tukey's Honestly Significant Difference (HSD) test was applied for pairwise comparisons among means.

## 3 Results and Discussion

### 3.1 Extraction Yield and Phytochemical Content of NLE

The extraction yield of NLE was 49.20% ± 1.78% (Table 1), indicating that nearly half of the dry plant material was recoverable as crude extract. Flórez et al. (2022) reported a higher extraction yield, ranging between 21.84% and 91.83%, when water was used as a solvent. In contrast, Zeković et al. (2017) and Fayza et al. (2025) reported lower yields of 3.66%, 1.50%, 8.75%, and 28.6%, respectively, when 96% ethanol, ethyl acetate, and pure methanol were used as extraction solvents. These variations in nettle leaf extraction yields were attributed to differences in the plant's harvesting season, maturity, and drying methods, which influence the concentration of polyphenols and other secondary metabolites (Fayza et al. 2025; Flórez et al. 2022; Ghaima et al. 2013). In this study, the obtained yield represents a typical range for polar solvent extractions of leafy plant matrices (Lohvina et al. 2022).

**TABLE 1:** Extraction yield, TPC, TFC, and antioxidant capacity (ABTS, DPPH, and FRAP) of nettle leaf extract.

Extraction yield (%)	Total phenolic content (mg GAE/g)	Total flavonoid content (mg QE/g)	DPPH	IC_50_ (μg/mL)	ABTS	IC_50_ (μg/mL)	FRAP
			TEAC (μmol Trolox/mL)		TEAC (μmol Trolox/mL)		Fe(II)/g
49.20 ± 1.78	81.62 ± 4.32	32.74 ± 0.98	21.96 ± 0.76	63.65 ± 0.45	17.51 ± 0.90	38.99 ± 0.62	5.93 ± 0.65

*Note:* Results are expressed as mean ± standard deviation.

NLE contained total phenolic content of 81.62 ± 4.32 mg GAE/g and a total flavonoid content of 32.74 ± 0.98 mg quercetin equivalents (QE)/g extract (Table 1). These values are consistent with previous reports (Adhikari et al. 2016; Flórez et al. 2022; Sawalha et al. 2025) but lower than those reported by Elez Garofulić et al. (2021); TPC ranged between 359.51 ± 14.58 and 2368.89 ± 30.11 mg GAE/100 g. Correlating with previous studies (Elez Garofulić et al. 2021; Sahal et al. 2025; Zhang et al. 2023), the results indicated a rich profile in phenolics and flavonoids, particularly caffeic acid derivatives, flavonol glycosides, and quercetin, which could contribute to the strong antimicrobial potential of NLE (Zhang et al. 2023; Yang et al. 2025).

Furthermore, the antioxidant activity, as assessed by radical scavenging assays, were 21.96 ± 0.76 μmol Trolox/mL for DPPH, 17.51 ± 0.90 μmol Trolox/mL for ABTS and 5.93 Fe(II)/g dry weight for FRAP (Table 1). The IC_50_ for DPPH was 63.65 ± 0.45 μg/mL and 38.99 ± 0.62 μg/mL for ABTS, confirming a moderated to high antioxidant capacity to neutralize free radicals. Similar results have been reported for nettle leaves (Flórez et al. 2022), pomegranate and guava leaf (Gaber et al. 2023) and *Spirulina platensis* (Shalaby and Shanab 2013) extracts. NLE antioxidant efficacy can be attributed to the synergistic action of multiple polyphenols, which are known to donate hydrogen atoms and stabilize reactive oxygen species (Gonelimali et al. 2018; Singh et al. 2012). These antioxidant properties can contribute to the stabilization of food matrices by delaying oxidative processes during storage as well as confer potential health benefits in humans (Sahal et al. 2025; Zhang et al. 2023).

### 3.2 In Vitro Cytotoxicity of NLE

**FIGURE 1:**
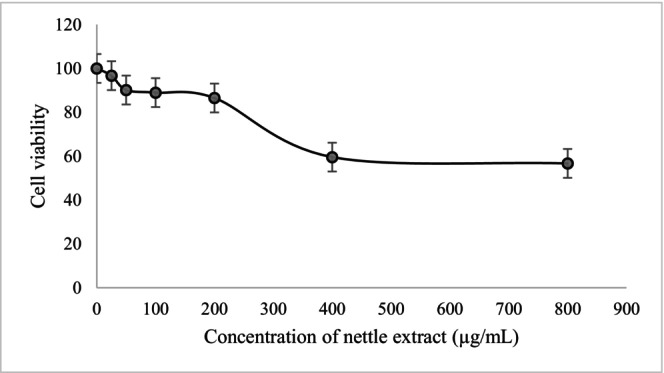
Effect of NLE on L929 mouse fibroblast cells (ATCC CCL‐1).

The effect of NLE on L929 fibroblast cells is presented in Figure 1. Across the tested concentrations (25–800 μg/mL), NLE exhibited low cytotoxicity, with cell viability remaining 85% at concentrations ≤ 200 μg/milk A dose‐dependent decline in viability became evident at ≥ 400 μg/mL, and a more pronounced reduction was observed at 800 μg/mL, where viability decreased to approximately 57.68%. The viability pattern observed here aligns with studies evaluating other culinary herbs used as natural preservatives, which typically maintain fibroblast viability above 75% at concentrations relevant to food systems (de Lima Pena et al. 2025). Also, nonlinear regression identified an estimated IC_50_ of 635.89 μg/mL. These findings are consistent with previous reports showing that *U. dioica* leaf extracts demonstrate high biocompatibility and low toxicity toward normal mammalian cells (Gül et al. 2012; Wójciak et al. 2024). For instance, D'Abrosca et al. (2019) reported that *U. dioica* L. decreased the proliferation of non‐small cell lung cancer H460, H1299, A549 and H322 cell lines in a time and dose‐dependent manner but showed no toxic effects on normal lung cells. Wójciak et al. (2024) demonstrated that concentration ranges of 25–200 μg/mL of both ethanol extract and polyphenolic fraction of *U. dioica* had no negative impact on the viability and metabolism of normal colon epithelial cells after 24 h of incubation. This low cytotoxicity has been attributed to the extract's antioxidant phenolics, flavonoids, vitamins, and trace minerals, which may protect cells rather than damage them (Flórez et al. 2022; Kőszegi et al. 2019; Xie et al. 2022). According to the National Cancer Institute (NCI) guidelines for crude plant extracts: IC_50_ ≤ 20 μg/mL indicates highly cytotoxic/very potent, 20–100 μg/mL, moderate cytotoxicity and > 100 μg/mL, weak or negligible cytotoxicity (Canga et al. 2022). Thus, indicating that NLE possesses low mammalian cytotoxicity within the concentration range, a promising attribute in food applications (Elez Garofulić et al. 2021; Wójciak et al. 2024; Zhang et al. 2023).

### 3.3 Antimicrobial Effect of NLE

**FIGURE 2:**
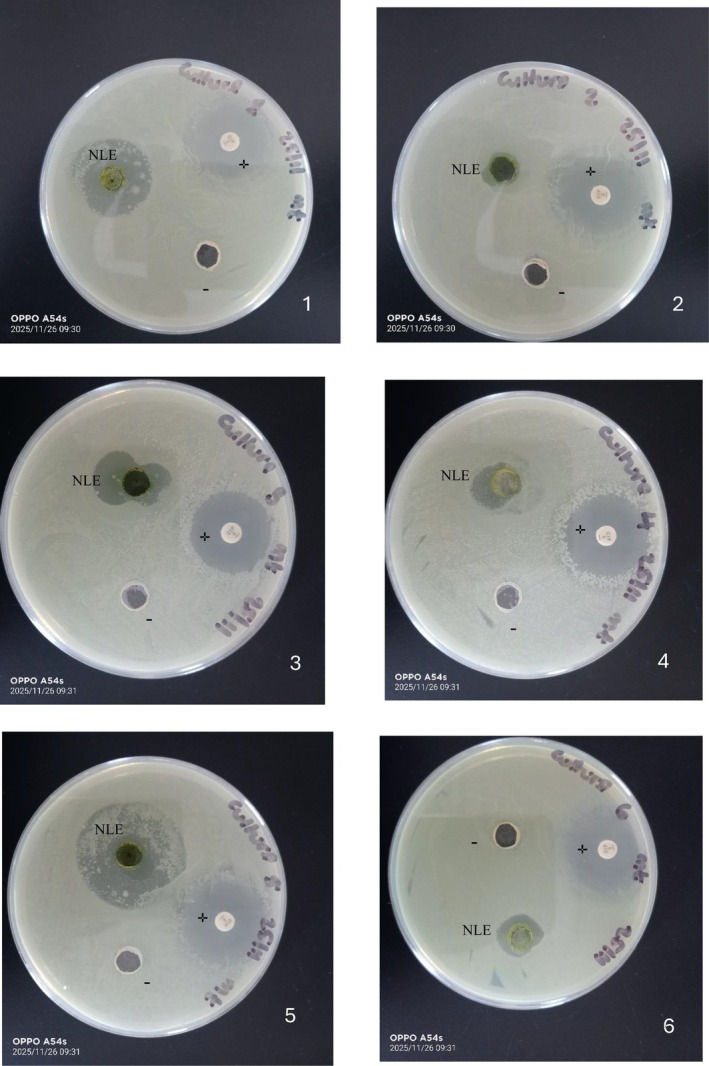
Agar well diffusion of selected foodborne pathogens: 1: *Staphylococcus aureus* NCTC 8532, 2: *Pseudomonas aeruginosa* ATCC 9027, 3: *Escherichia coli* NCTC 9001, 4: *Listeria monocytogenes* NCTC 10357, 5: *Bacillus cereus* NCTC 2599, and 6: *Salmonella enterica* serovar Typhimurium NCTC 74. NLE: Nettle leaf extract, −: Negative control (deionized water), +: Positive control (Ciprofloxacin 5 μg).

NLE demonstrated measurable inhibitory activity against all six tested foodborne pathogens (Figure 2). Its mean inhibition zones ranged from 2 ± 0.71 ( *P. aeruginosa* ) to 32 ± 0.45 mm ( *B. cereus* ) at 100 mg/mL of NLE (Table 2), indicating broad‐spectrum antimicrobial potential. Also, MIC and MBC ranged between 10.01–89.38 and 11.21–52.50 μg/mL respectively. Rahmani et al. (2024) reported similar MIC and MBC values ranging between 11.25–95 mg./mL and 11.25–190 μg/mL respectively. While all pathogens were inhibited, susceptibility differed markedly; Gram‐positive bacteria (*B. cereus, S. aureus* and *L. monocytogenes* ) displayed the highest sensitivity, Gram negative ( *E. coli* and *S. typhimurium*) exhibited moderate sensitivity, reflecting the protective role of the Gram‐negative outer membrane, which can limit penetration of hydrophobic phenolic compounds (Harrison et al. 2022; Zenão et al. 2017). The smallest zones (2 mm) were observed for *P. aeruginosa* , a microorganism inherently resistant to many plant extracts due to efflux pumps, biofilm‐forming capacity, and membrane‐level resistance mechanism (Lemoni et al. 2025; Oulahal and Degraeve 2022). These results agree with earlier reports (Elez Garofulić et al. 2021; Ghaima et al. 2013; Gülçin et al. 2004; Oulahal and Degraeve 2022) that nettle extracts exert strong antimicrobial effects against Gram‐positive organisms through disruption of membrane permeability and interference with cellular energy pathways.

**TABLE 2:** Inhibition zone, MIC and MBC of NLE.

Organisms tested	NLE (50 mg/mL)	Inhibition zone (mm)		MIC (μg/mL)	MBC (μg/mL)
		NLE (100 mg/mL)	Ciprofloxacin (5 μg.)		
*S. aureus*	12 ± 0.25	25 ± 0.98	14 ± 0.60	62.28	42.10
*L. monocytogenes*	10 ± 0.44	17 ± 0.66	13 ± 0.21	11.92	20.33
*E. coli*	13.46 ± 0.88	18 ± 0.60	14 ± 0.04	17.36	11.21
*S. enterica* serovar Typhimurium	16 ± 0.54	20 ± 0.31	15 ± 0.28	30.95	14.56
*B. cereus*	15 ± 0.64	32 ± 0.45	14 ± 0.69	89.38	52.50
*P. aeruginosa*	0.4 ± 0.06	2 ± 0.71	13 ± 0.88	10.01	13.18

In contrast to low toxicity toward mammalian cells (Figure 1), NLE showed clear antimicrobial activity against *S. aureus, L. monocytogenes, E. coli, S. typhimurium, B. cereus*, and *P. aeruginosa* in line with previous studies (Harrison et al. 2022; Kukric et al. 2012; Zenão et al. 2017). Plant extracts with high phenolic content often exhibit this dual characteristic, where microbial membranes are more susceptible to disruption than mammalian cells (de Lima Pena et al. 2025; Elez Garofulić et al. 2021). NLE exhibited antimicrobial activity primarily due to its phenolic compounds, flavonoids, and other phytochemicals (Elez Garofulić et al. 2021; Harrison et al. 2022; Sahal et al. 2025). Phenolics such as caffeic acid, chlorogenic acid, and quercetin derivatives, commonly found in nettle, exert antimicrobial activity by increasing membrane permeability, inducing oxidative stress, and interfering with microbial energy systems (Elez Garofulić et al. 2021; Oulahal and Degraeve 2022; Sahal et al. 2025), mechanisms that are generally less harmful to eukaryotic cells due to their robust antioxidant and repair pathways. Their broad antimicrobial effect suggests potential use as a natural preservative to extend the shelf‐life of food products (Alirezalu et al. 2021; Tanyitiku et al. 2024). For instance, ready‐to‐eat potato products are highly susceptible to microbial spoilage due to their high moisture, moderate pH, and rich carbohydrate content, which support growth of *Bacillus*, *Pseudomonas*, and Enterobacteriaceae under chilled conditions (Doan and Davidson 2000; Lauridsen and Knøchel 2003; Thomas et al. 2002). As such, incorporating nettle extract into the mashed potato matrix could therefore delay microbial proliferation, particularly for Gram‐positive pathogens commonly associated with cooked starchy foods.

### 3.4 Selectivity Index

The selectivity index (SI) provides a critical indicator of the balance between antimicrobial efficacy and mammalian cell safety and is increasingly used to evaluate plant‐derived antimicrobials for food applications (Famuyide et al. 2019). In the present study, NLE showed high SI values against all tested foodborne and spoilage microorganisms, ranging from 7.11 to 63.54 (Table 3). The strongest selectivity was observed against *L. monocytogenes* (SI = 53.36) and *P. aeruginosa* (SI = 63.54), two organisms that play a major role in the spoilage and safety of refrigerated mashed potatoes (Doan and Davidson 2000; Thomas and Masters 1988). This high selectivity explains the pronounced reduction in psychrotrophic counts observed in NLE‐treated samples during cold storage (Figure 6). The susceptibility of *L. monocytogenes* to NLE is consistent with the known vulnerability of Gram‐positive bacteria to phenolic acids and flavonoids that disrupt cytoplasmic membrane integrity and inhibit energy metabolism (Famuyide et al. 2019; Gonelimali et al. 2018).

**TABLE 3:** Selectivity index (SI) of NLE against selected foodborne pathogens.

Organisms	*S. aureus*	*L. monocytogenes*	*E. coli*	*S. enterica* serovar Typhimurium	*B. cereus*	*P. aeruginosa*
SI	10.21	53.36	36.63	20.55	7.11	63.54

High SI values were also observed for *E. coli* (36.63) and *Salmonella Typhimurium* (20.55), supporting the significant suppression of Enterobacteriaceae in treated mashed potatoes (Figure 7). Although Gram‐negative bacteria possess an outer membrane that typically limits penetration of phytochemicals (De Rossi et al. 2025; Oulahal and Degraeve 2022), polyphenols present in nettle leaves (e.g., quercetin, caffeic acid, chlorogenic acid) are known to interfere with efflux pumps, metal homeostasis, and oxidative stress regulation, enhancing antimicrobial action (Gülçin et al. 2004; Sahal et al. 2025). Comparably, *Bacillus cereus* exhibited the lowest SI value (7.11), consistent with the well‐documented resilience of spore‐forming bacteria. Nonetheless, this value is still considered acceptable for food‐preservation applications, particularly when used in conjunction with refrigeration or other hurdle technologies (Karnwal and Malik 2024; Lemoni et al. 2025). The reduction of *B. cereus* counts in treated mashed potatoes (Figure 8) is therefore consistent with the extract's moderate but effective selectivity against this pathogen. With this, the SI findings provide mechanistic support for the observed shelf‐life extension of mashed potatoes treated with NLE, demonstrating that antimicrobial effects occurred at concentrations well below those causing cytotoxicity.

### 3.5 Shelf‐Life Stability of NLE‐Incorporated Mashed Potatoes

#### 3.5.1 Changes in Moisture, pH and Water Activity During Storage

Monitoring moisture content, pH, and water activity is critical for determining the shelf‐life and safety of mashed potatoes, as these parameters collectively influence microbial growth, enzymatic activity, and physicochemical stability during storage. High moisture levels create a favorable environment for spoilage microorganisms and accelerate chemical reactions, while pH affects the growth potential of bacteria, yeasts, and molds, with lower pH generally inhibiting pathogenic species. Water activity (a_w_), which reflects the availability of free water for microbial metabolism, is a key predictor of microbial proliferation and oxidative changes.

**FIGURE 3:**
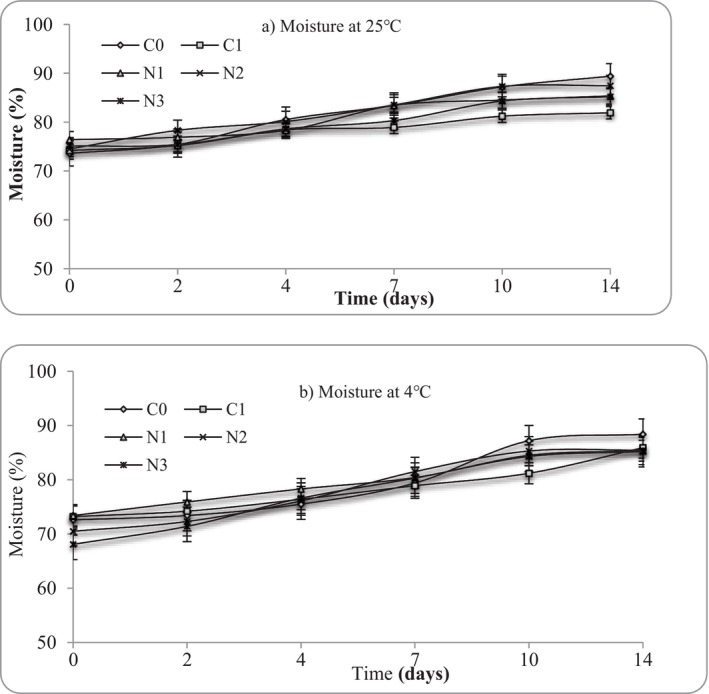
Moisture variation at (a) 25°C and (b) 4°C in mashed potato samples. C0 (negative control): No preservative, C1 (positive control): 0.025% (w/v) nisin, N1: 0.5% (w/v) nettle leaf extract, N2: 1.0% (w/v) nettle leaf extract, and N3: 2.0% (w/v) nettle leaf extract.

**FIGURE 4:**
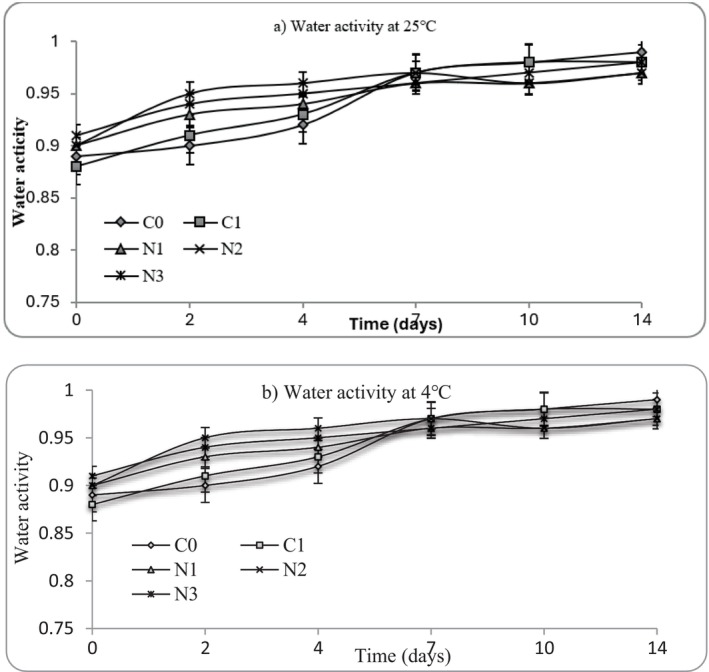
Water activity changes at (a) 25°C and (b) 4°C in mashed potato samples. C0 (negative control): No preservative, C1 (positive control): 0.025% (w/v) nisin, N1: 0.5% (w/v) nettle leaf extract, N2: 1.0% (w/v) nettle leaf extract, and N3: 2.0% (w/v) nettle leaf extract.

In the current study, mashed potato samples storage at both 4°C and 25°C exhibited a gradual increase in moisture content and water activity (a_w_) from Day 0 to Day 14, regardless of nettle extract incorporation (Figures 3 and 4). Regarding moisture, the mashed potatoes exhibited high moisture content from Day 0 (> 68%), consistent with the intrinsic water‐rich nature (typically between 75% and 80%) of cooked potatoes (do Nascimento et al. 2020). Water activity (a_w_) values remained high (0.89–0.99) across all treatments and storage conditions, reflecting the moist, starch‐rich nature of mashed potatoes. This behavior is consistent with previous reports on cooked starch‐based foods, where post‐processing structural relaxation of gelatinised starch granules and partial breakdown of the potato cell matrix promote water migration and redistribution within the product (do Nascimento et al. 2020). As storage progressed, starch retrogradation and weakening of the gel network can release previously bound water, resulting in increased measurable moisture and a_w_ values (Jantrawut et al. 2023). The increase in water activity may also be attributed to enzymatic and microbial activity, which can liberate low‐molecular‐weight compounds and free water, particularly under refrigerated and ambient storage conditions (FAO 2003; Fernández et al. 2009; Yu et al. 2024). Previous reports have shown that spoilage microorganism growth accelerates the release of free water due to enzymatic disruption of starch‐protein networks (Zhang et al. 2023; Yu et al. 2024). Although nettle extract possesses antimicrobial properties, its incorporation did not completely inhibit these physicochemical changes, suggesting that structural and biochemical processes intrinsic to mashed potatoes dominate water dynamics during storage.

**FIGURE 5:**
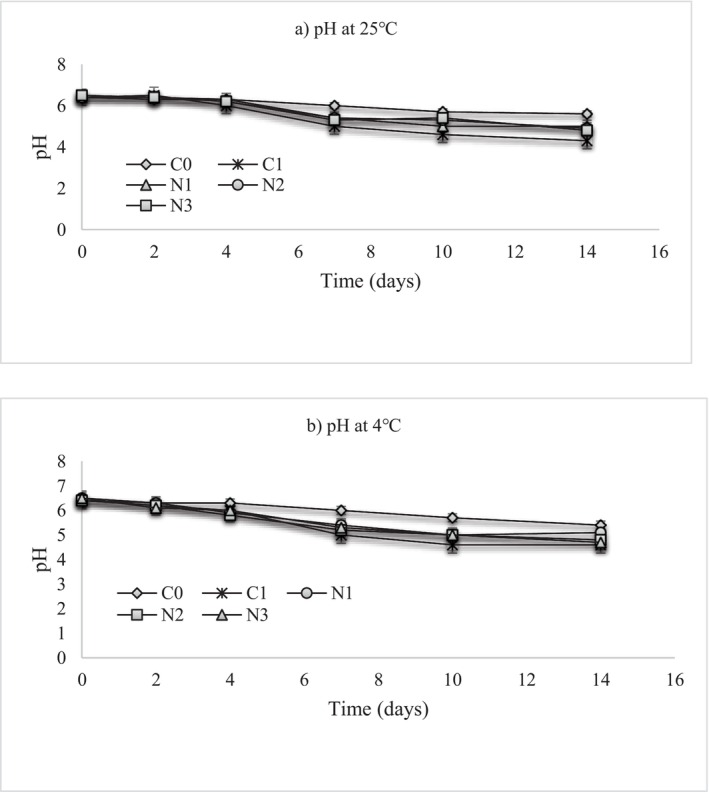
pH changes at (a) 25°C and (b) 4°C in mashed potato samples. C0 (negative control): No preservative, C1 (positive control): 0.025% (w/v) nisin, N1: 0.5% (w/v) nettle leaf extract, N2: 1.0% (w/v) nettle leaf extract, and N3: 2.0% (w/v) nettle leaf extract.

In contrast to moisture and a_w_, a progressive decrease in pH was observed over the same storage period (Figure 5). With the addition of NLE producing no significant shift in pH, initial pH values of the mashed potatoes were mildly acidic (approximately 6.0–6.3), consistent with typical values for cooked tubers, whose natural pH ranges between 5.5 and 6.5 (Fernández et al. 2009). During storage, a slight decrease in pH was observed in nettle‐treated samples, particularly by Day 7. The untreated samples exhibited a slower rate of pH decline, indicating partial suppression of acid‐producing microflora. This pH decline is typical for mashed potato products and has been associated with the metabolic activity of psychrotrophic spoilage microorganisms and lactic acid‐producing bacteria (Lauridsen and Knøchel 2003; Doan and Davidson 2000). Similar trends have been reported in refrigerated potato purées and other cooked vegetable products, where acidification occurs even in the presence of natural antimicrobial agents (Lauridsen and Knøchel 2003; Lemoni et al. 2025; Thomas et al. 2002). Typically, pH levels below 4.6 are detrimental to the survival of microorganisms and bacteria tend to live within a narrower pH range (4.5–9.0) than yeasts or molds (2.0–10.0) (Lima et al. 2023).

Despite high moisture and a_w_, a lower pH stability observed in nettle‐treated samples reflects the extract's ability to moderate both biochemical and microbiological changes. This pattern aligns with earlier findings where polyphenol‐rich extracts inhibited enzymatic and bacterial processes associated with pH decline and water release in chilled mashed vegetable products (Yang et al. 2025). As such, understanding these parameters enables the development of clean‐label preservation approaches, where natural antimicrobials and processing techniques can be optimized to maintain product stability without reliance on synthetic additives.

#### 3.5.2 Microbial Growth

**FIGURE 6:**
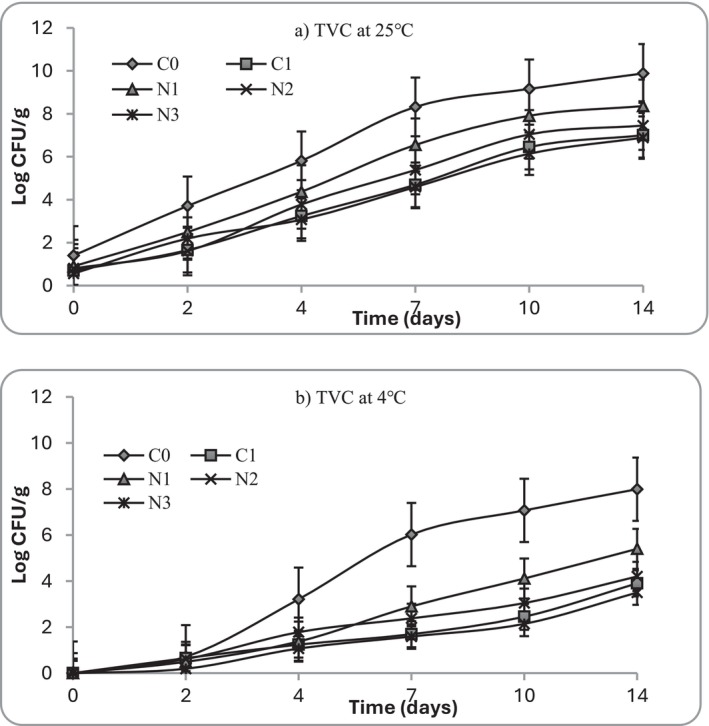
Total viable count (TVC) at (a) 25°C and (b) 4°C in mashed potato samples. C0 (negative control): No preservative, C1 (positive control): 0.025% (w/v) nisin, N1: 0.5% (w/v) nettle leaf extract, N2: 1.0% (w/v) nettle leaf extract, and N3: 2.0% (w/v) nettle leaf extract.

**FIGURE 7:**
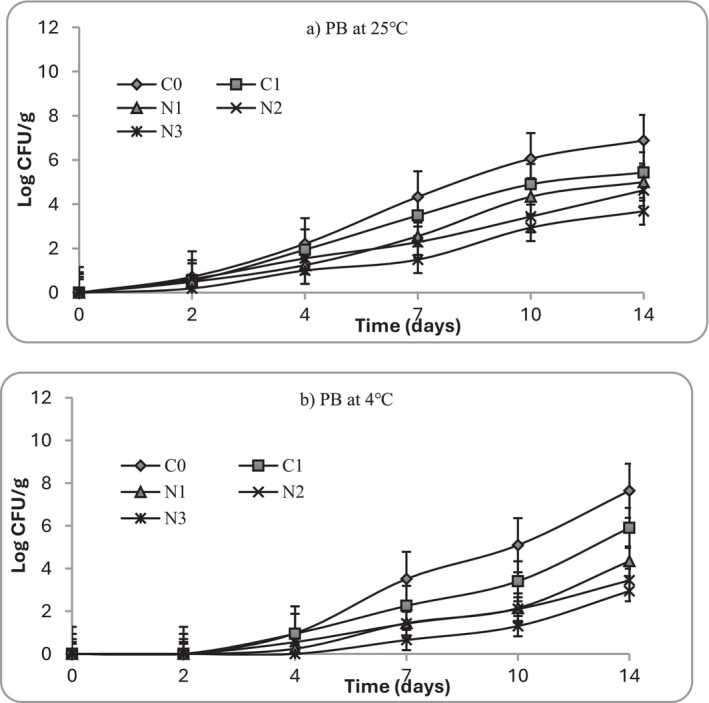
Growth of psychrotrophic bacteria (PB) at (a) 25°C and (b) 4°C in mashed potato samples. C0 (negative control): No preservative, C1 (positive control): 0.025% (w/v) nisin, N1: 0.5% (w/v) nettle leaf extract, N2: 1.0% (w/v) nettle leaf extract, and N3: 2.0% (w/v) nettle leaf extract.

At Day 0, all mashed potato samples showed low initial microbial loads (< 2 Log_10_ CFU/g), confirming hygienic preparation (Figure 6). During storage at 25°C, untreated controls (C0) exhibited rapid microbial proliferation, exceeding the spoilage threshold of 7 Log_10_ CFU/g by Day 4. Similar growths were observed in fresh‐cut potatoes stored between 0°C and 10°C, where the total number of colonies of samples showed a straight upward trend, ranging from 2.5–3 Log_10_ CFU/mL to 4.4–5.5 Log_10_ CFU/mL (Zhao et al. 2022). Psychrotrophs, important for refrigerated foods, increased steadily in C0 samples during cold storage, surpassing 6 Log_10_ CFU/g by Day 10 (Figure 7). In contrast, samples treated with nettle extracts demonstrated concentration‐dependent inhibition: N1 delayed spoilage until Day 5, while N2 and N3 extended shelf life to 7 and 9 days, respectively. The nisin control (C1) showed comparable suppression to N3, maintaining total aerobic count below 6 Log_10_ CFU/g until Day 7 (Figure 6). In terms of psychrotrophic growth, nettle treatments, especially N2 and N3, suppressed more effectively than nisin (C1), with counts remaining < 5 Log_10_ CFU/g at Day 14. Under refrigerated storage (4°C), microbial growth was significantly slowed across all treatments, particularly in the first 2 days during sample storage. However, C0 still reached spoilage limits by Day 10, whereas N3 and C1 remained within acceptable limits (< 7 Log_10_ CFU/g) for the entire 14‐day study period. These results align with previous findings (Lauridsen and Knøchel 2003) where the number of mesophilic bacteria in raw pre‐peeled potatoes stored at 4°C was found to display a lag phase before an increase was observed between Days 5 and 7. Moreover, the retail shelf life of pre‐peeled potatoes was estimated at only 6 days when stored at or below 4°C (Doan and Davidson 2000). The current findings are consistent with previous research that mashed potatoes are highly susceptible to microbial contamination when exposed to unfavorable storage temperature and post‐processing environments (Lauridsen and Knøchel 2003; Thomas et al. 2002). Also, it indicates the significance of plant‐derived phenolics to inhibit mesophilic and psychrotrophic microorganisms (Petcu et al. 2023).

**FIGURE 8:**
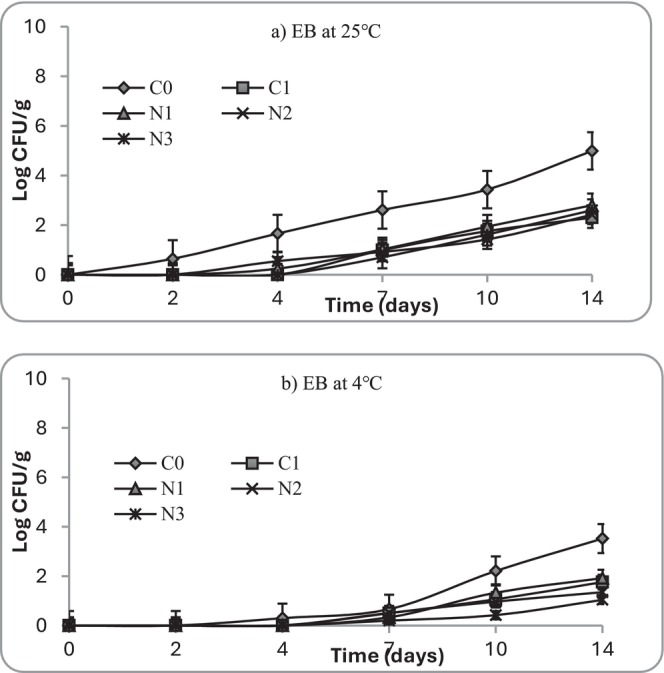
Growth of Enterobacteriaceae (EB) at (a) 25°C and (b) 4°C in mashed potato samples. C0 (negative control): No preservative, C1 (positive control): 0.025% (w/v) nisin, N1: 0.5% (w/v) nettle leaf extract, N2: 1.0% (w/v) nettle leaf extract, and N3: 2.0% (w/v) nettle leaf extract.

Enterobacteriaceae count, indicative of Gram‐negative spoilage and hygiene quality, was detectable in the control (C0) right from Day 2 and it became highest by Day 14, reaching 4.9 Log_10_ CFU/g at 25°C (Figure 8). In contrast, Enterobacteriaceae were markedly reduced in the nettle‐ and nisin‐treated samples, remaining below 3 Log_10_ CFU/g in N3 and C2. Nettle extracts indicated a significant reduction in these counts in a dose‐dependent manner, with the highest concentration reducing Enterobacteriaceae to < 2 Log_10_ CFU/g by Day 7. The selective inhibition of Enterobacteriaceae was consistent with the antibacterial spectrum of Gram‐negative bacteria by NLE (See Table 2). For the identification tests, four bacteria including *Enterobacter cloacae, Enterobacter kobei, Escherichia coli*, and *Serratia liquefaciens* were identified in both samples stored at 25°C and 4°C. These organisms are often introduced through environmental contamination, post harvesting and/or during handling and can thrive in moist, nutrient‐rich foods like mashed potatoes (Gonelimali et al. 2018; Gu et al. 2022).

**FIGURE 9:**
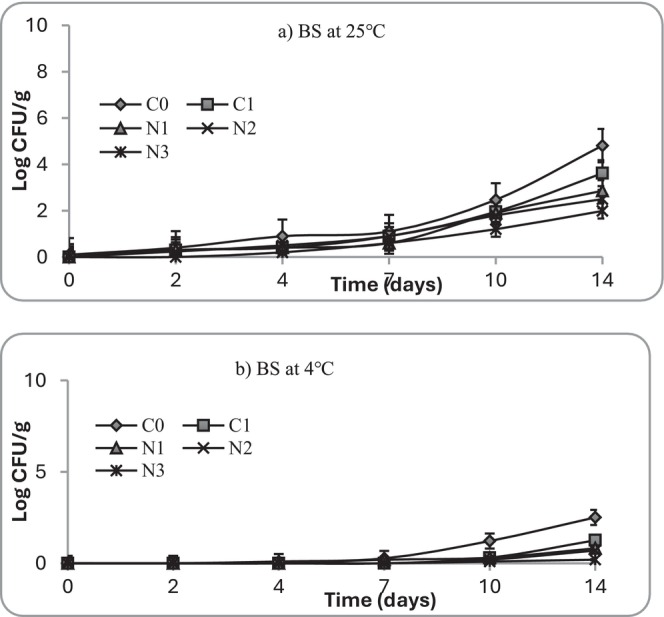
Growth of *Bacillus* spp. (BS) at (a) 25°C and (b) 4°C in mashed potato samples. C0 (negative control): No preservative, C1 (positive control): 0.025% (w/v) nisin, N1: 0.5% (w/v) nettle leaf extract, N2: 1.0% (w/v) nettle leaf extract, and N3: 2.0% (w/v) nettle leaf extract.

The findings indicated that heat‐resistant *Bacillus* spores survived during processing and increased during storage (Figure 9). The outgrowth of *Bacillus cereus* was particularly evident in C0 and C1 at ambient conditions, whereas nettle extract (N2, N3) significantly suppressed spore germination and vegetative growth, a finding consistent with the activity of polyphenolic compounds against spore‐forming bacteria (Karnwal and Malik 2024). The inhibition of *Bacillus* spores is particularly notable because these organisms are responsible for both spoilage and potential foodborne illness in potato products (Doan and Davidson 2000). For example, after 5–12 days of storage at 25°C, 60% of pasteurized potato purees were found to contain *B. cereus*, and by Day 20, 80% of the samples stored at 10°C were found to contain this pathogen (Thomas et al. 2002). *B. cereus* strains were also found in pureed containing broccoli, carrots, zucchini, and split peas in the range of 6–8 Log10 CFU/g at 20°C and 4–6 Log10 CFU/g at 10°C (Choma et al. 2000; Thomas et al. 2002). At counts of ca 10^8^/g of sample, Thomas and Masters (1988) predominantly isolated *Bacillus*, *Streptococcus*, and *Staphylococcus* in precooked potato‐topped pies stored at 4°C and 37°C, indicating such food products represented a potential public health risk if stored at inappropriate temperatures.

**FIGURE 10:**
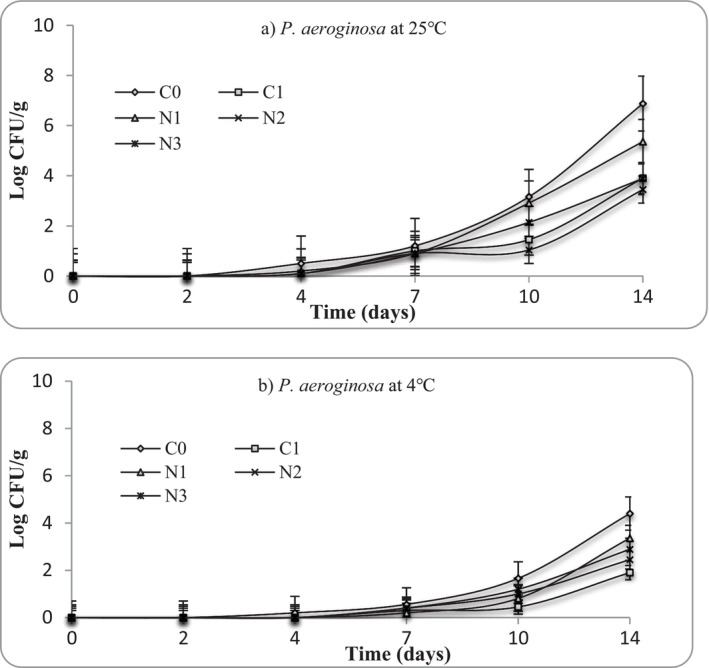
Growth of *Pseudomonas aeruginosa* at (a) 25°C and (b) 4°C in mashed potato samples. C0 (negative control): No preservative, C1 (positive control): 0.025% (w/v) nisin, N1: 0.5% (w/v) nettle leaf extract, N2: 1.0% (w/v) nettle leaf extract, and N3: 2.0% (w/v) nettle leaf extract.

**FIGURE 11:**
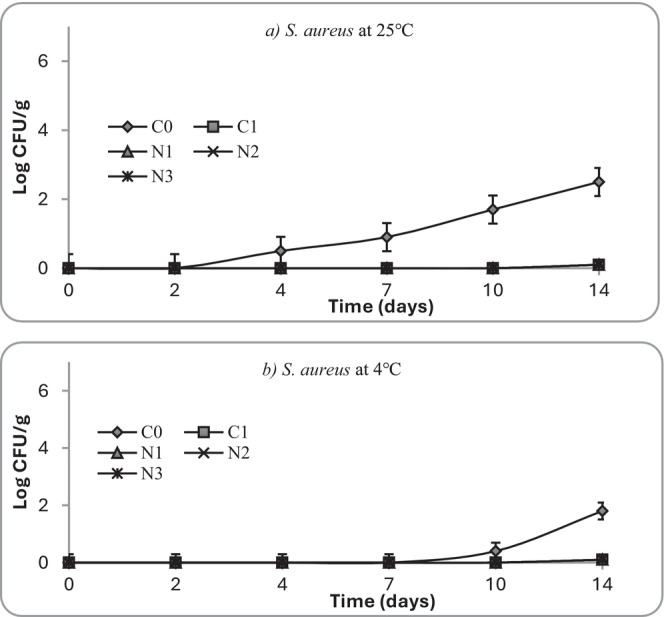
Growth of *Staphylococcus aureus* at (a) 25°C and (b) 4°C in mashed potato samples. C0 (negative control): No preservative, C1 (positive control): 0.025% (w/v) nisin, N1: 0.5% (w/v) nettle leaf extract, N2: 1.0% (w/v) nettle leaf extract, and N3: 2.0% (w/v) nettle leaf extract.

*Pseudomonas aeruginosa* was low in all samples and was not detected until Day 4 at 25°C of storage (Figure 10). By Day 14, C0 was 6.7 Log_10_ CFU/g and 4.2 Log_10_ CFU/g at 25°C and 4°C respectively. Similarly, Lauridsen and Knøchel (2003) enumerated *Pseudomonas* spp. in raw pre‐peeled potatoes packed in modified atmosphere and stored at 4°C (3.5 × 10^5^ CFU/g) and 15°C (710 CFU/g) for 7 days. In line with *Pseudomonas* spp., *S. aureus* growth was mostly enumerated in C0, and by Day 14, it reached 2.3 Log_10_CFU/g under ambient and 1.9 Log_10_ CFU/g at refrigerated storage, whereas it remained undetectable in nisin (C1) and nettle‐incorporated samples (N1‐N3) (Figure 11). While mashed potatoes are not a high‐risk food for *S. aureus* under proper hygiene, the results indicated that NLE provided additional safety assurance, supporting its use as a natural antimicrobial.

**FIGURE 12:**
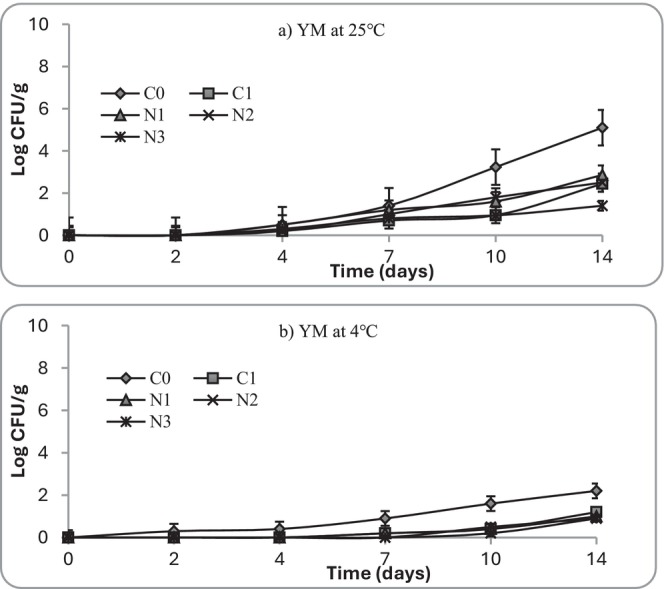
Growth of yeast and molds (YM) at (a) 25°C and (b) 4°C in mashed potato samples. C0 (negative control): No preservative, C1 (positive control): 0.025% (w/v) nisin, N1: 0.5% (w/v) nettle leaf extract, N2: 1.0% (w/v) nettle leaf extract, and N3: 2.0% (w/v) nettle leaf extract.

Furthermore, yeasts and molds were detected by Day 4 in C0 stored at 25°C, reaching > 3 Log_10_ CFU/g by Day 7, and could be correlated with visible spoilage (Figure 12). Fungal counts increased gradually in control samples, reaching 4 Log_10_ CFU/g after 5 days at ambient storage. At 4°C, fungal counts remained < 3 Log_10_ CFU/g in all samples, though control samples eventually showed surface colonization after Day 12. Using DRBC agar enabled more selective enumeration of yeasts and molds, showing that nettle extracts (N2, N3) restricted fungal growth almost as effectively as nisin. Nettle‐ and nisin‐treated samples limited fungal growth, with counts remaining < 2.5 Log_10_ CFU/g during storage. Refrigerated storage slowed fungal proliferation in all groups. The results indicate that nettle extract inhibited yeast and molds, likely due to the combined antimicrobial and antioxidant activity.

Overall, the microbial results demonstrate that nettle extracts, particularly at 1.0%–2.0% (w/v), are effective in suppressing microorganisms and extending the shelf life of mashed potatoes. NLE antimicrobial efficacy was comparable to that of commercial nisin, especially under refrigerated conditions, as well as other commonly used herbs including rosemary, oregano (Gonelimali et al. 2018; Lemoni et al. 2025; Oulahal and Degraeve 2022). These inhibitory effects are largely attributable to the synergistic action of phenolic acids (caffeic and chlorogenic acid) and flavonoids (quercetin derivatives), which disrupt microbial membranes, interfere with enzyme function, and delay spore germination (Elez Garofulić et al. 2021; Harrison et al. 2022; López‐Hortas et al. 2020; Sahal et al. 2025; Zenão et al. 2017). Importantly, nettle‐treated samples showed reduced growth of psychrotrophs *B. cereus* and *S. aureus* , which are major public health concerns in potato‐based ready‐to‐eat foods (Doan and Davidson 2000; Thomas et al. 2002).

#### 3.5.3 Instrumental Taste

**FIGURE 13:**
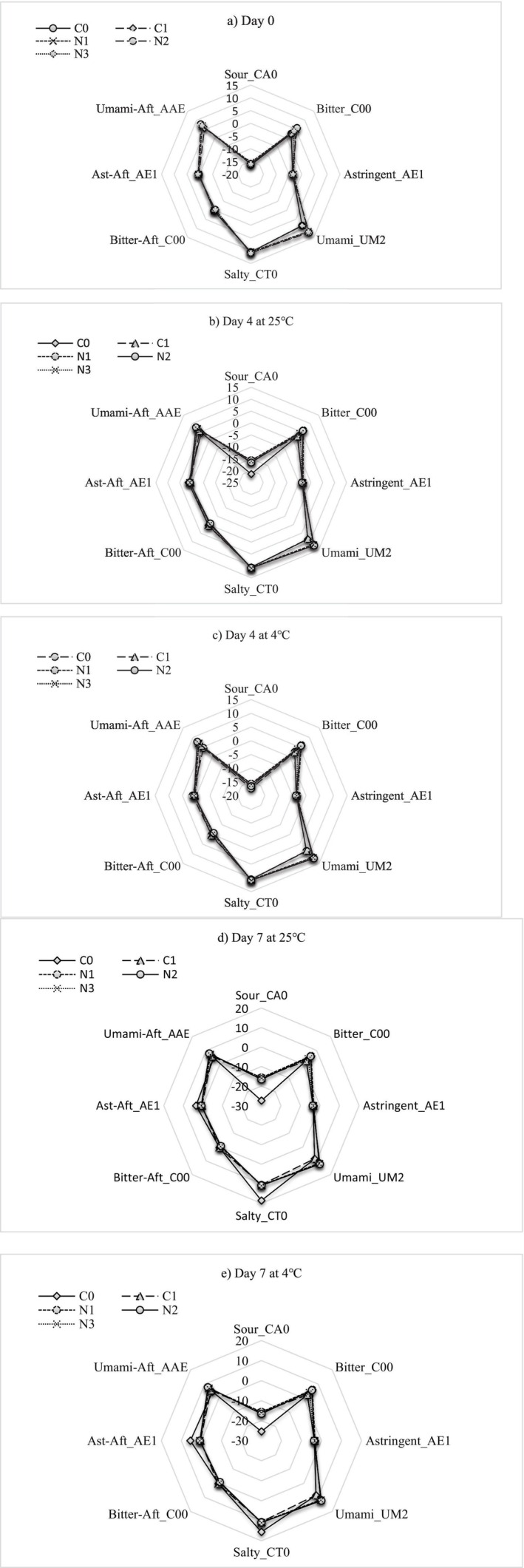
Electronic tongue analysis of mashed potatoes at (a) Day 0, (b) Day 4 at 25°C, (c) Day 4 at 4°C, (d) Day 7 at 25°C, (e) Day 7 at 4°C. C0 (negative control): No preservative, C1 (positive control): 0.025% (w/v) nisin, N1: 0.5% (w/v) nettle leaf extract, N2: 1.0% (w/v) nettle leaf extract, and N3: 2.0% (w/v) nettle leaf extract.

E‐tongue analysis provided quantitative sensor outputs for sourness, saltiness, bitterness, umami, and aftertaste indices for Day 0, 4, and 7 (Figure 13). At Day 0, control mashed potatoes were dominated by umami and mild saltiness (Figure 13a), consistent with the typical taste characteristics of polysaccharides, amino acids, and added milk or butter components in cooked potato (Thomas and Masters 1988; Xu et al. 2023). Also, in Figure 13a, the nettle‐treated samples exhibited slightly higher bitterness and astringency scores, attributable to phenolic compounds such as caffeic acid, ferulic acid, and quercetin derivatives, naturally abundant in *Urtica dioica* extracts (Kozlowska et al. 2019; Mahmoudi et al. 2014; Tanyitiku and Njombissie Petcheu 2025). By Day 4, the separation between clusters slightly increased, as the control samples showed the first signs of sensory deterioration (Figure 13b). Elevated sourness and bitter aftertaste signals were observed, likely resulting from the accumulation of organic acids and breakdown metabolites due to early microbial spoilage and enzymatic degradation (Fayaz et al. 2024; Fernández et al. 2009). In contrast, the treated samples maintained a more stable sensory profile, reflecting the antimicrobial efficacy of nettle extract (Elez Garofulić et al. 2021; Harrison et al. 2022; Sahal et al. 2025; Sengun et al. 2020; Zenão et al. 2017).

By Day 7, the contrast of C0 was even more pronounced particularly at 25°C of storage (Figure 13d). The control samples shifted significantly toward sourness and bitterness sensory attributes, strongly associated with spoilage in starchy foods during cold storage (Fayaz et al. 2024). Meanwhile, the treated samples still maintained closer proximity to their Day 4 attributes (Figure 13c), with only marginal increases in bitterness and astringency. The attenuated taste drift is consistent with the antimicrobial findings of this study (Section 3.3), where nettle extract effectively suppressed the growth of spoilage organisms such as *Pseudomonas*, *Enterobacteriaceae*, and *Bacillus* spp. These microbes are among the major contributors to early sensory deterioration in cooked potato products and ready‐to‐eat products (Doan and Davidson 2000). For instance, *Bacillus* species can cause spoilage problems such as “ropiness” in bread and “bittiness” in milk (UKHSA 2024). Notably, total viable count of > 10^6^ CFU/g reported in this research was associated with the acceptability and organoleptic quality of certain foods (UKHSA 2024). The antioxidant activity of nettle extract may also stabilize flavor‐active lipid and phenolic compounds during storage, resulting in greater preservation of the original taste profile (Tanyitiku and Njombissie Petcheu 2025; Zhang et al. 2023). Reduced drift in aftertaste sensors further supports the hypothesis that nettle extract slows spoilage‐related biochemical reactions. Similar outcomes have been documented in vegetable purees and potato‐based products supplemented with natural phenolic extracts, where antioxidant and antimicrobial effects contribute to prolonged sensory stability (Doan and Davidson 2000; Thomas and Masters 1988; Thomas et al. 2002).

## 4 Conclusion

To the best of our knowledge, this study represents one of the first systematic investigations into the application of nettle ( *Urtica dioica* L.) extracts as natural antimicrobial agents for extending the shelf life of mashed potato products, in line with current clean‐label and sustainable food preservation strategies. The extracts, rich in phenolic acids and flavonoids, exhibited strong antioxidant capacity and broad‐spectrum antimicrobial activity, with Gram‐positive bacteria (*Bacillus cereus, Staphylococcus aureus*, and *Listeria monocytogenes* ) showing greater susceptibility than Gram‐negative pathogens, likely due to structural differences in cell wall composition. When incorporated into mashed potatoes, nettle extracts significantly suppressed the growth of total viable bacteria, psychrotrophs, Enterobacteriaceae, *B. cereus, S. aureus, Pseudomonas aeruginosa*, and spoilage fungi under both refrigerated and ambient storage conditions. Although higher extract concentrations introduced mild bitterness and astringency, these sensory effects were less pronounced than the spoilage‐associated off‐flavors observed in untreated controls, indicating an overall improvement in product stability and quality. Future research should focus on optimizing extract dosage to balance antimicrobial efficacy and sensory acceptance, exploring synergistic combinations with other natural preservation hurdles, and validating performance under industrial‐scale processing and extended storage conditions.

## Author Contributions

**Saritha Kagula:** data curation, validation, methodology, investigation, visualization, writing – original draft, writing – review and editing. **Steven Harte:** data curation, investigation, validation, writing – review and editing. **Srivathsa Kumbaji:** data curation, validation, methodology, writing – review and editing. **Rania Harastani:** data curation, validation, writing – review and editing. **Mary Nkongho Tanyitiku:** data curation, validation, supervision, resources, funding acquisition, writing – original draft, writing – review and editing All authors reviewed the manuscript.

## Funding

The authors have nothing to report.

## Ethics Statement

The authors have nothing to report.

## Consent

The authors have nothing to report.

## Conflicts of Interest

The authors declare no conflicts of interest.

## Data Availability

Data will be made available on request.

## References

[fsn371551-bib-0001] Adhikari, B. M. , A. Bajracharya , and A. K. Shrestha . 2016. “Comparison of Nutritional Properties of Stinging Nettle ( *Urtica dioica* ) Flour With Wheat and Barley Flours.” Food Science & Nutrition 4, no. 1: 119–124. 10.1002/fsn3.259. 26788318 PMC4708629

[fsn371551-bib-0002] Ahmadi, M. , V. Razavilar , A. Motallebi , R. Kenari , and A. A. Khanipour . 2014. “Effects of Hydroalcoholic and Water Extracts of Nettle Leaf (*Urtica dioica* L.) on Chemical Properties of Superchilled Minced Meat of Common Kilka (*Clupeonella cultriventris* Caspia).” Journal of Food Quality and Hazards Control 1: 85–88.

[fsn371551-bib-0003] Alirezalu, K. , M. Yaghoubi , Z. Nemati , and B. Farmani . 2021. “Efficacy of Stinging Nettle Extract in Combination With ε‐Polylysine on the Quality, Safety, and Shelf Life of Rainbow Trout Fillets.” Food Science & Nutrition 9: 1542–1550. 10.1002/fsn3.2129. 33747468 PMC7958555

[fsn371551-bib-0004] Arfa, N. , M. A. M. Zandi , and S. M. Hassan . 2023. “Effect of Bioactive Compounds of Sting Nettle (*Urtica diocia* l.) Leaves on Shelf Life of the Salty Biscuits.” Food Technology Research Journal 2, no. 3: 73–85. 10.21608/ftrj.2023.333790.

[fsn371551-bib-0005] Canga, I. , P. Vita , A. I. Oliveira , M. Á. Castro , and C. Pinho . 2022. “In Vitro Cytotoxic Activity of African Plants: A Review.” Molecules 27, no. 15: 4989. 10.3390/molecules27154989. 35956938 PMC9370645

[fsn371551-bib-0006] Cao, H. , M. Huang , Z. Liu , et al. 2025. “Influencing the Retrogradation Properties of Potato Mash Through Multi‐Scale Structural Modifications Mediated by Enzymatic Hydrolysis Forms.” Food Chemistry 485: 144467. 10.1016/j.foodchem.2025.144467. 40306064

[fsn371551-bib-0007] Choma, C. , M. H. Guinebretière , F. Carlin , et al. 2000. “Prevalence, Characterization and Growth of *Bacillus cereus* in Commercial Cooked Chilled Foods Containing Vegetables.” Journal of Applied Microbiology 88, no. 4: 617–625. 10.1046/j.1365-2672.2000.00998.x. 10792519

[fsn371551-bib-0008] CIP . 2024. Potato Facts and Figures. International Potato Center. https://cipotato.org/potato/potato‐facts‐and‐figures/.

[fsn371551-bib-0009] CLSI . 2020. Performance Standards for Antimicrobial Susceptibility Testing. 30th Edition. Clinical and Laboratory Standard Institute.

[fsn371551-bib-0010] D'Abrosca, B. , V. Ciaramella , V. Graziani , et al. 2019. “ *Urtica dioica* L. Inhibits Proliferation and Enhances Cisplatin Cytotoxicity in NSCLC Cells via Endoplasmic Reticulum‐Stress Mediated Apoptosis.” Scientific Reports 9, no. 1: 4986. 10.1038/s41598-019-41372-1. 30899059 PMC6428841

[fsn371551-bib-0011] de Lima Pena, F. , M. C. de Souza , V. L. Sanches , et al. 2025. “Phenolic Profile, Bioactivity and Cytotoxicity of Plant Extracts From Thyme, Ginger, Garlic, Ground Roasted Coffee and Coffee Silverskin.” International Journal of Food Properties 28, no. 1: 2519843. 10.1080/10942912.2025.2519843.

[fsn371551-bib-0012] De Rossi, L. , G. Rocchetti , L. Lucini , and A. Rebecchi . 2025. “Antimicrobial Potential of Polyphenols: Mechanisms of Action and Microbial Responses—A Narrative Review.” Antioxidants 14: 200. 10.3390/antiox14020200. 40002386 PMC11851925

[fsn371551-bib-0013] do Nascimento, R. F. , M. H. G. Canteri , S. Á. Rodrigues , and J. L. Kovaleski . 2020. “Use of Sodium Metabisulphite and Ascorbic Acid to Control Browning in Ready‐To‐Eat Processed Potatoes During Prolonged Storage.” Potato Research 63, no. 4: 615–625. 10.1007/s11540-020-09461-1.

[fsn371551-bib-0014] Doan, C. H. , and P. M. Davidson . 2000. “Microbiology of Potatoes and Potato Products: A Review.” Journal of Food Protection 63, no. 5: 668–683. 10.4315/0362-028X-63.5.668. 10826729

[fsn371551-bib-0015] Elez Garofulić, I. , V. Malin , M. Repajić , et al. 2021. “Phenolic Profile, Antioxidant Capacity and Antimicrobial Activity of Nettle Leaves Extracts Obtained by Advanced Extraction Techniques.” Molecules 26, no. 20: 6153. 10.3390/molecules26206153. 34684733 PMC8538125

[fsn371551-bib-0016] Famuyide, I. M. , A. O. Aro , F. O. Fasina , J. N. Eloff , and L. J. McGaw . 2019. “Antibacterial and Antibiofilm Activity of Acetone Leaf Extracts of Nine Under‐Investigated South African Eugenia and Syzygium (Myrtaceae) Species and Their Selectivity Indices.” BMC Complementary and Alternative Medicine 19: 141. 10.1186/s12906-019-2547-z. 31221162 PMC6587284

[fsn371551-bib-0017] FAO . 2003. “Handling and Preservation of Fruits and Vegetables by Combined Methods for Rural Areas.” In Technical Manual FAO Agricultural Services Bulletin, vol. 149. Food And Agriculture Organization of the United Nations.

[fsn371551-bib-0018] Fattahi, S. , E. Zabihi , Z. Abedian , et al. 2014. “Total Phenolic and Flavonoid Contents of Aqueous Extract of Stinging Nettle and In Vitro Antiproliferative Effect on Hela and BT‐474 Cell Lines.” International Journal of Molecular and Cellular Medicine 3, no. 2: 102–107. 25035860 PMC4082812

[fsn371551-bib-0019] Fayaz, U. , S. Srivastava , A. H. Dar , et al. 2024. “Recent Insights Into e‐Tongue Interventions in Food Processing Applications: An Updated Review.” Current Food Science and Technology Reports 2, no. 2: 169–182. 10.1007/s43555-024-00028-6.

[fsn371551-bib-0020] Fayza, K. , B. Malika , F. Meriem , B. Moussa , B. Djilali , and B. Amel . 2025. “LC‐MS/MS Analysis, Antimicrobial and Antioxidant Potential of Phenolic Extracts Derived From *Urtica dioica* Leaves and Roots.” Pakistan Journal of Pharmaceutical Sciences 38, no. 5: 1528–1538. 10.36721/pjps.2025.38.5.reg.13072.1. 40996168

[fsn371551-bib-0021] FDA (Food and Drug Administration) . 1988. Nisin Preparation. Affirmation of GRAS Status as a Direct Human Food Ingredient. Vol. 53, 11247. Food and Drug Administration Fed. Regist.

[fsn371551-bib-0022] Fernández, C. , W. Canet , and M. Dolores Alvarez . 2009. “The Effect of Long‐Term Frozen Storage on the Quality of Frozen and Thawed Mashed Potatoes With Added Cryoprotectant Mixtures.” International Journal of Food Science and Technology 44, no. 7: 1373–1387. 10.1111/j.1365-2621.2009.01967.x.

[fsn371551-bib-0023] Flórez, M. , P. Cazón , and M. Vázquez . 2022. “Antioxidant Extracts of Nettle ( *Urtica dioica* ) Leaves: Evaluation of Extraction Techniques and Solvents.” Molecules 27, no. 18: 6015. 10.3390/molecules27186015. 36144748 PMC9500655

[fsn371551-bib-0024] Gaber, N. B. , S. I. El‐Dahy , and E. A. Shalaby . 2023. “Comparison of ABTS, DPPH, Permanganate, and Methylene Blue Assays for Determining Antioxidant Potential of Successive Extracts From Pomegranate and Guava Residues.” Biomass Conversion and Biorefinery 13, no. 5: 4011–4020. 10.1007/s13399-021-01386-0.

[fsn371551-bib-0025] Ghaima, K. , N. M. Hashim , and S. A. Ali . 2013. “Antibacterial and Antioxidant Activities of Ethyl Acetate Extract of Nettle (*Urtica dioica*) and Dandelion (*Taraxacum officinale*).” Journal of Applied Pharmaceutical Science 3: 96–99. 10.7324/JAPS.2013.3518.

[fsn371551-bib-0026] Gonelimali, F. D. , J. Lin , W. Miao , et al. 2018. “Antimicrobial Properties and Mechanism of Action of Some Plant Extracts Against Food Pathogens and Spoilage Microorganisms.” Frontiers in Microbiology 9: 1639. 30087662 10.3389/fmicb.2018.01639 PMC6066648

[fsn371551-bib-0027] Gu, Q. , P. Li , H. Jiang , et al. 2022. “Foodborne Pathogens of Enterobacteriaceae, Their Detection and Control.” In Enterobacteria, edited by S. B. Bhardwaj . IntechOpen.

[fsn371551-bib-0028] Gül, S. , B. Demirci , K. H. C. Başer , H. A. Akpulat , and P. Aksu . 2012. “Chemical Composition and In Vitro Cytotoxic, Genotoxic Effects of Essential Oil From *Urtica dioica* L.” Bulletin of Environmental Contamination and Toxicology 88, no. 5: 666–671. 10.1007/s00128-012-0535-9. 22310841

[fsn371551-bib-0029] Gülçin, İ. , Ö. İ. Küfrevioǧlu , M. Oktay , and M. E. Büyükokuroǧlu . 2004. “Antioxidant, Antimicrobial, Antiulcer and Analgesic Activities of Nettle (*Urtica dioica* L.).” Journal of Ethnopharmacology 90, no. 2: 205–215. 10.1016/j.jep.2003.09.028. 15013182

[fsn371551-bib-0030] Gülhan, B. , and F. Yangilar . 2024. “Effects of Nettle (*Urtica dioica*) Extract on Versus Pathogenic Microorganisms in Yogurt Production.” Journal of Tekirdag Agricultural Faculty 21, no. 3: 759–770. 10.33462/jotaf.1368617.

[fsn371551-bib-0031] Harrison, F. , J. Furner‐Pardoe , and E. Connelly . 2022. “An Assessment of the Evidence for Antibacterial Activity of Stinging Nettle ( *Urtica dioica* ) Extracts.” Access Microbiology 4, no. 3: 000336. 10.1099/acmi.0.000336. 35693473 PMC9175978

[fsn371551-bib-0032] Jantrawut, P. , K. Buri , O. Chambin , W. Ruksiriwanich , Y. Phimolsiripol , and T. Chaiwarit . 2023. “Rheological Properties, Printability and Microstructure of Buttermilk‐Mashed Potatoes Incorporated With Chlorpheniramine Maleate as a Material for 3D Food Printing.” International Journal of Food Science and Technology 58, no. 11: 5796–5808. 10.1111/ijfs.16683.

[fsn371551-bib-0033] Karnwal, A. , and T. Malik . 2024. “Exploring the Untapped Potential of Naturally Occurring Antimicrobial Compounds: Novel Advancements in Food Preservation for Enhanced Safety and Sustainability.” Frontiers in Sustainable Food Systems 8: 1307210.

[fsn371551-bib-0034] Kőszegi, K. , E. Békássy‐Molnár , N. Koczka , T. Kerner , and É. Stefanovits‐Bányai . 2019. “Changes in Total Polyphenol Content and Antioxidant Capacity of Stinging Nettle (*Urtica dioica* L.) From Spring to Autumn.” Periodica Polytechnica, Chemical Engineering 64: 548–554. 10.3311/PPch.14338.

[fsn371551-bib-0035] Kőszegi, K. , G. Végvári , É. Stefanovits‐Bányai , E. Békássy‐Molnár , and A. Maraz . 2023. “Influence of the Harvesting Seasons on the Polyphenol Composition and Antimicrobial Activity of Stinging Nettle (*Urtica dioica* L.) Extracts.” Acta Alimentaria 52: 589–600. 10.1556/066.2023.00156.

[fsn371551-bib-0036] Kozlowska, M. , A. Zbikowska , K. Marciniak‐Lukasiak , and M. Kowalska . 2019. “Herbal Extracts Incorporated Into Shortbread Cookies: Impact on Color and Fat Quality of the Cookies.” Biomolecules 9, no. 12: 858. 10.3390/biom9120858. 31835857 PMC6995587

[fsn371551-bib-0037] Kramer, B. , J. Thielmann , A. Hickisch , P. Muranyi , J. Wunderlich , and C. Hauser . 2015. “Antimicrobial Activity of Hop Extracts Against Foodborne Pathogens for Meat Applications.” Journal of Applied Microbiology 118, no. 3: 648–657. 10.1111/jam.12717. 25494620

[fsn371551-bib-0038] Kregiel, D. , E. Pawlikowska , and H. Antolak . 2018. “ *Urtica* spp.: Ordinary Plants With Extraordinary Properties.” Molecules 23, no. 7: 1664. 10.3390/molecules23071664. 29987208 PMC6100552

[fsn371551-bib-0039] Kukric, Z. , L. Topalić‐Trivunović , B. Kukavica , et al. 2012. “Characterization of Antioxidant and Antimicrobial Activities of Nettle Leaves (*Urtica dioica* L.).” Acta Periodica Technologica 43: 257–272. 10.2298/APT1243257K.

[fsn371551-bib-0040] Lauridsen, L. , and S. Knøchel . 2003. “Microbiological Stability and Diversity in Raw Pre‐Peeled Potatoes Packed in Different Atmospheres.” European Food Research and Technology 217, no. 5: 421–426. 10.1007/s00217-003-0787-z.

[fsn371551-bib-0041] Leffler, T. P. , C. R. Moser , B. J. McManus , et al. 2008. “Determination of Moisture and Fat in Meats by Microwave and Nuclear Magnetic Resonance Analysis: Collaborative Study.” Journal of AOAC International 91, no. 4: 802–810. 10.1093/jaoac/91.4.802. 18727540

[fsn371551-bib-0042] Lemoni, Z. , K. Evangeliou , T. Lymperopoulou , and D. Mamma . 2025. “Incorporation of Edible Plant Extracts as Natural Food Preservatives: Green Extraction Methods, Antibacterial Mechanisms and Applications.” Food 14, no. 23: 4000. 10.3390/foods14234000. PMC12691971 41375938

[fsn371551-bib-0043] Lima, V. , C. A. Pinto , and J. A. Saraiva . 2023. “The Dependence of Microbial Inactivation by Emergent Nonthermal Processing Technologies on pH and Water Activity.” Innovative Food Science & Emerging Technologies 89: 103460.

[fsn371551-bib-0044] Lohvina, H. , M. Sándor , and M. Wink . 2022. “Effect of Ethanol Solvents on Total Phenolic Content and Antioxidant Properties of Seed Extracts of Fenugreek (*Trigonella foenum‐graecum* L.) Varieties and Determination of Phenolic Composition by HPLC‐ESI‐MS.” Diversity 14, no. 1: 7. 10.3390/d14010007.

[fsn371551-bib-0045] López‐Hortas, L. , C. Le Juge , E. Falqué , H. Domínguez , and M. D. Torres . 2020. “Bioactive Extracts From Edible Nettle Leaves Using Microwave Hydrodiffusion and Gravity and Distillation Extraction Techniques.” Process Biochemistry 94: 66–78. 10.1016/j.procbio.2020.04.012.

[fsn371551-bib-0046] Mahmoudi, R. , K. Amini , O. Fahri , and M. Alem . 2014. “Aroma Profile and Antimicrobial Properties of Alcoholic and Aqueous Extracts From Root, Leaf and Stalk of Nettle (*Urtica dioica* L.).” Journal of Microbiology, Biotechnology and Food Sciences 4: 220–224. 10.15414/jmbfs.2014-15.4.3.220-224.

[fsn371551-bib-0047] Mei, J. , X. Ma , and J. Xie . 2019. “Review on Natural Preservatives for Extending Fish Shelf Life.” Food 8, no. 10: 1–23. 10.3390/foods8100490. PMC6835557 31614926

[fsn371551-bib-0048] Modarresi‐Chahardehi, A. , D. Ibrahim , S. Fariza‐Sulaiman , and L. Mousavi . 2012. “Screening Antimicrobial Activity of Various Extracts of *Urtica dioica* .” Revista de Biología Tropical 60, no. 4: 1567–1576. 10.15517/rbt.v60i4.2074. 23342511

[fsn371551-bib-0049] Oulahal, N. , and P. Degraeve . 2022. “Phenolic‐Rich Plant Extracts With Antimicrobial Activity: An Alternative to Food Preservatives and Biocides?” Frontiers in Microbiology 12: 753518. 35058892 10.3389/fmicb.2021.753518 PMC8764166

[fsn371551-bib-0050] Petcu, C. D. , D. Tăpăloagă , O. D. Mihai , et al. 2023. “Harnessing Natural Antioxidants for Enhancing Food Shelf Life: Exploring Sources and Applications in the Food Industry.” Food 12, no. 17: 3176. 10.3390/foods12173176. PMC10486681 37685108

[fsn371551-bib-0051] Rahmani, Z. , M. Karimi , I. Saffari , H. Mirzaei , M. Nejati , and R. Sharafati Chaleshtori . 2024. “Nanoemulsion and Nanoencapsulation of a Hydroethanolic Extract of Nettle (*Urtica dioica*) and Wormwood (*Artemisia absinthium*): Comparison of Antibacterial and Anticancer Activity.” Frontiers in Chemistry 12: 1266573. 10.3389/fchem.2024.1266573. 38292020 PMC10824895

[fsn371551-bib-0052] Sahal, A. , A. Hussain , S. Kumar , et al. 2025. “Nettle (*Urtica dioica*) Leaves as a Novel Food: Nutritional, Phytochemical Profiles, and Bioactivities.” Food Chemistry: X 28: 102607. 10.1016/j.fochx.2025.102607. 40520698 PMC12167447

[fsn371551-bib-0053] Sawalha, H. , G. Qalalweh , H. Nazzal , A. Kmail , and I. Qoraan . 2025. “Antibacterial Potential of Plant Extracts Against Foodborne Pathogenic Bacteria: A Phytochemical and Bioactive Analysis.” Journal of Pure and Applied Microbiology 19, no. 3: 2211–2226. 10.22207/JPAM.19.3.48.

[fsn371551-bib-0054] Sengun, I. Y. , A. Kirmizigul , K. Atlama , and B. Yilmaz . 2020. “The Viability of *Lactobacillus rhamnosus* in Orange Juice Fortified With Nettle (*Urtica dioica* L.) and Bioactive Properties of the Juice During Storage.” LWT 118: 108707. 10.1016/j.lwt.2019.108707.

[fsn371551-bib-0055] Shalaby, E. , and S. Shanab . 2013. “Comparison of DPPH and ABTS Assays for Determining Antioxidant Potential of Water and Methanol Extracts of *Spirulina platensis* .” Indian Journal of Marine Sciences 42: 556–564.

[fsn371551-bib-0056] Šic Žlabur, J. , S. Radman , N. Opacic , et al. 2022. “Application of Ultrasound as Clean Technology for Extraction of Specialized Metabolites From Stinging Nettle ( *Urtica dioica* L.).” Frontiers in Nutrition 9: 870923. 10.3389/fnut.2022.870923. 35669064 PMC9165585

[fsn371551-bib-0057] Singh, D. R. , S. Dar , and D. P. Sharma . 2012. “Antibacterial Activity and Toxicological Evaluation of Semi Purified Hexane Extract of *Urtica dioica* Leaves.” Research Journal of Medicinal Plant 6: 123–135. 10.3923/rjmp.2012.123.135.

[fsn371551-bib-0058] Tanyitiku, M. N. , P. Bessem , and I. C. Njombissie Petcheu . 2024. “Gluten‐Free Corn Cookies Incorporated With Stinging Nettle Leaf Flour: Effect on Physical Properties, Storage Stability, and Health Benefits.” International Journal of Food Science 2024: 1–13. 10.1155/2024/8864560. PMC11319057 39135739

[fsn371551-bib-0059] Tanyitiku, M. N. , and I. C. Njombissie Petcheu . 2025. “Technofunctional Properties of Stinging Nettle (*Urtica dioica* L.) Leaf Flour and Its Enhancing Pasting, Physical and Sensory Characteristics in Gluten‐Free Rice Waffles.” Journal of Food Quality 2025: 9418554. 10.1155/jfq/9418554.

[fsn371551-bib-0060] Thaipong, K. , U. Boonprakob , K. Crosby , L. Cisneros‐Zevallos , and D. Hawkins . 2006. “Comparison of ABTS, DPHH, FRAP, and ORAC Assays for Estimating Antioxidant Activity From Guava Fruit Extracts.” Journal of Food Composition and Analysis 19: 669–675.

[fsn371551-bib-0061] Thomas, C. J. , and F. Masters . 1988. “Microbial Spoilage of Pre‐Cooked Potato‐Topped Pies.” Journal of Applied Bacteriology 64, no. 3: 227–234. 10.1111/j.1365-2672.1988.tb03379.x. 3133345

[fsn371551-bib-0062] Thomas, L. V. , R. E. Ingram , H. E. Bevis , E. A. Davies , C. F. Milne , and J. Delves‐Broughton . 2002. “Effective Use of Nisin to Control *Bacillus* and *Clostridium* Spoilage of a Pasteurized Mashed Potato Product.” Journal of Food Protection 65, no. 10: 1580–1585. 10.4315/0362-028x-65.10.1580. 12380742

[fsn371551-bib-0063] UKHSA . 2024. Guidelines for Assessing the Microbiological Safety of Ready‐To‐Eat Foods Placed on the Market. Interpretation of Test Results Generated by UKHSA Food Water and Environmental Microbiology Services Laboratories. UK Health Security Agency. https://assets.publishing.service.gov.uk/media/66debd72e87ad2f1218265e1/UKHSA‐ready‐to‐eat‐guidelines2024.pdf.

[fsn371551-bib-0064] Wójciak, M. , R. Paduch , P. Drozdowski , et al. 2024. “Antioxidant and Anti‐Inflammatory Effects of Nettle Polyphenolic Extract: Impact on Human Colon Cells and Cytotoxicity Against Colorectal Adenocarcinoma.” Molecules 29, no. 21: 5000. 10.3390/molecules29215000. 39519642 PMC11547774

[fsn371551-bib-0065] Xie, J. , S. D. Robinson , E. K. Gilding , et al. 2022. “Neurotoxic and Cytotoxic Peptides Underlie the Painful Stings of the Tree Nettle (*Urtica ferox*).” Journal of Biological Chemistry 298, no. 8: 1–12. 10.1016/j.jbc.2022.102218. PMC9352542 35780839

[fsn371551-bib-0066] Xu, J. , Y. Li , L. Kaur , J. Singh , and F. Zeng . 2023. “Functional Food Based on Potato.” Food 12: 2145. 10.3390/foods12112145. PMC10253093 37297391

[fsn371551-bib-0067] Yang, Y. , J. Liu , X. Wang , J. Deng , and H. Jiang . 2025. “Effects of Different Polyphenols on the Structural, Physicochemical, Digestive and 3D Printing Properties of Mashed Potatoes.” Food Chemistry 488: 144919. 10.1016/j.foodchem.2025.144919. 40446652

[fsn371551-bib-0068] Younes, M. , P. Aggett , F. Aguilar , et al. 2017. “Scientific Opinion on the Safety of Nisin (E 234) as a Food Additive in the Light of New Toxicological Data and the Proposed Extension of Use. EFSA Panel on Food Additives and Nutrient Sources Added to Food (ANS).” EFSA Journal 15, no. 12: 5063. 10.2903/j.efsa.2017.5063. PMC7009836 32625365

[fsn371551-bib-0069] Yu, Y. , W. Weng , Z. Ren , Y. Zhang , P. P. Li , and L. Shi . 2024. “Quality Deterioration of Mashed Potatoes During the Freeze‐Thaw Cycle: From the Perspective of Moisture and Microstructure.” Food Chemistry: X 23: 101753. 10.1016/j.fochx.2024.101753. 39280215 PMC11402148

[fsn371551-bib-0070] Zeković, Z. , A. Cvetanović , J. Švarc‐Gajić , et al. 2017. “Chemical and Biological Screening of Stinging Nettle Leaves Extracts Obtained by Modern Extraction Techniques.” Industrial Crops and Products 108: 423–430. 10.1016/j.indcrop.2017.06.055.

[fsn371551-bib-0071] Zenão, S. , A. Aires , C. Dias , M. J. Saavedra , and C. Fernandes . 2017. “Antibacterial Potential of *Urtica dioica* and *Lavandula angustifolia* Extracts Against Methicillin Resistant *Staphylococcus aureus* Isolated From Diabetic Foot Ulcers.” Journal of Herbal Medicine 10: 53–58. 10.1016/j.hermed.2017.05.003.

[fsn371551-bib-0072] Zhang, Y. , X. Zhang , M. H. Zafar , et al. 2023. “Research Progress in Physiological Effects of Resistant Substances of *Urtica dioica* L. on Animal Performance and Feed Conversion.” Frontiers in Plant Science 14: 1164363. 37448866 10.3389/fpls.2023.1164363 PMC10336547

[fsn371551-bib-0073] Zhao, S. , X. Han , B. Liu , et al. 2022. “Shelf‐Life Prediction Model of Fresh‐Cut Potato at Different Storage Temperatures.” Journal of Food Engineering 317: 110867.

